# Diversity and Chemical Characterization of Apple (*Malus* sp.) Pollen: High Antioxidant and Nutritional Values for Both Humans and Insects

**DOI:** 10.3390/antiox13111374

**Published:** 2024-11-09

**Authors:** Milica M. Fotirić Akšić, Mirjana B. Pešić, Ilinka Pećinar, Aleksandra Dramićanin, Danijel D. Milinčić, Aleksandar Ž. Kostić, Uroš Gašić, Mihajlo Jakanovski, Marko Kitanović, Mekjell Meland

**Affiliations:** 1 Faculty of Agriculture, University of Belgrade, Nemanjina 6, 11000 Belgrade, Serbia; fotiric@agrif.bg.ac.rs (M.M.F.A.); mpesic@agrif.bg.ac.rs (M.B.P.); ilinka@agrif.bg.ac.rs (I.P.); danijel.milincic@agrif.bg.ac.rs (D.D.M.); akostic@agrif.bg.ac.rs (A.Ž.K.); marko.kitanovic@agrif.bg.ac.rs (M.K.); 2 Faculty of Chemistry, University of Belgrade, Studentski Trg 12-16, 11000 Belgrade, Serbia; akosovic@chem.bg.ac.rs; 3 Institute for Biological Research “Siniša Stanković”, University of Belgrade, Bulevar Despota Stefana 142, 11060 Belgrade, Serbia; uros.gasic@ibiss.bg.ac.rs; 4 Innovative Centre of the Faculty of Chemistry, University of Belgrade, Studentski Trg 12-16, 11000 Belgrade, Serbia; jakanovski@chem.bg.ac.rs; 5 Norwegian Institute of Bioeconomy Research—NIBIO Ullensvang, Ullensvangvegen 1005, 5781 Lofthus, Norway

**Keywords:** *Malus domestica* Borkh., *Malus sylvestris* (L.) Mill., crab apple, sugar, protein, polyphenols, Raman

## Abstract

Pollen represents a reward for pollinators and is a key element in plant–insect interactions, especially in apples, which are entomophilous species and require cross-pollination to produce economically valuable yields. The aim of this study was to analyze the chemical content of the pollen in 11 apple cultivars (‘Red Aroma’, ‘Discovery’, ‘Summerred’, ‘Rubinstep’, ‘Elstar’, ‘Dolgo’, ‘Professor Sprenger’, ‘Asfari’, ‘Eden’, ‘Fryd’ and ‘Katja’) grown in Norway and try to establish a relationship between them and insect attractiveness. In the applied chemical analysis, 7 sugars and sugar alcohols, 4 organic acids, 65 phenolic compounds, 18 hydroxycinnamic acid amides (phenylamides), a large number of polypeptides with a molecular weight of 300 kDa to <6.5 kDa, lipids, carotenoids, starch, pectin and cellulose were determined. The crab apples ‘Dolgo’ and ‘Professor Sprenger’, which are used as pollenizers in commercial orchards, had the highest level of sucrose, total polyphenol content (prevent oxidative damages in insects), antioxidant capacity, hydroxybenzoic acids and derivatives, quercetin and derivatives, dihyrochalcone, epicatechin, putrescine derivates, and proteins with molecular weight 66–95 kDa and >95 kDa, which made them interesting for insect pollenizers. Only the pollen of the crab apples contained quercetin-3-*O*-(2″-*O*-malonyl)-hexoside, which can be used as a marker for the apple species *Malus sylvestris* (L.) Mill. Apple floral pollen is a rich source of bioactive components and can be used to prevent and/or cure diseases or can be included in diets as a “superfood”.

## 1. Introduction

Floral pollen is a powdery substance produced in the androecium of flowers during blooming through the processes of microsporogenesis and microgametogenesis. It plays a crucial role in the reproduction of these plants, as it contains the sperm cells necessary for double fertilizing the embryo sac, resulting in the production of a seed [1]. During flowering, the pollen grains are “mature” and fully capable/viable to perform ‘double’ fertilization. When they fall on a sticky stigma, pollen tubes begin to grow through the transmitting tissue of the pistil, and if the crossing combination is compatible, they reach the ovule and the embryo sac that contains the egg cell [2]. The pollen itself has different shapes, sizes, polarities, symmetries and exine sculptures depending on the species. The chemistry of pollen grains differs depending on their botanical and geographic origin, plant age, edaphic and ecologic parameters (temperature, soil, water, and light intensity) as well as their type of pollination and pollinators [3,4,5]. Depending on its different plant sources, pollen is reported to consist of about 200 compounds [6]. It is a rich source of proteins (10–40 g/100 g dry weight), lipids (1–13 g/100 g dry weight, mostly unsaturated fatty acids), carbohydrates (13–55 g/100 g dry weight, both polysaccharides and low molecular sugars), phenolics (0.2 and 2.5% of flavonoids, leukotrienes, catechins, phenolic acids, flavonol and flavonol glycosides), amino acids (mainly prolin and aspartic acid, glutamic acid, leucine, lysine and arginine which make up 2% of the total weight of the pollen grains), dietary fiber and pectin (0.3–20 g/100 g dry weight), ash (2–6 g/100 g dry weight), minerals (K, Mg, P, Ca, S, B, Zn, Cu, Mn, Cu, Mn), water- and oil-soluble vitamins (b-carotene, B1, B2, B3, B5, B6, C, biotin, folic acid, tocopherol) and up to 50% water [7,8,9,10,11].

Previous research showed that bee pollen has a high antioxidant capacity [12,13]. Due to its high nutritional value, especially in the form of phenolic acids, flavonoids, fatty acids, phytosterols, organic acids, enzymes, sterols, triterpenes, phytohormones and alkaloids, pollen is appreciated as a functional food or added to other foods, both for human use and for supplementing animal feed [14,15]. The daily consumption of pollen stimulates blood circulation, increases immunity, and enhances physical and mental activities, protects the liver, helps to improve the performance of the heart, exerts a positive effect on the hematopoietic system, protects against ischemic heart disease and strokes, increases insulin secretion and reduces blood sugar levels [15]. Pollen has antimicrobial, antiradiation, antioxidant, antifungal, hepatoprotective, chemoprotective, and/or anti-inflammatory effects [10]. Unfortunately, pollen causes various allergic reactions, such as rhinitis, conjunctivitis and asthma, which often occur simultaneously in the same patient during the pollen season [16].

Pollen is a key element in plant–insect interactions; it is a reward for the insects that pollinate [17]. Sometimes it may contain toxic compounds (alkaloids, some phenols, some sugars) that are used to repel herbivores and to defend the pollen against non-pollinators [18,19]. From a bee’s perspective, an adequate supply of pollen is imperative to continue all the stages of development in the hive, reproduction, brood rearing, body size, venom production, resistance to pathogens and pesticides, and to ensure its long-term survival [20,21,22].

The apple (*Malus* × *domestica* Borkh.) is a member of the family Rosaceae. Due to its large and diverse gene pool, successful production all over the world, different colors and sizes, desirable aroma and taste, good transportability and year-round storage, it is the most important fruit species in temperate zones worldwide. Apple production in Norway is on an upward trend, with the area of modern and high-density apple orchards currently around 1500 ha, with an annual output of over 12,000 tons [23]. It is the northernmost fruit tree-growing area in the world, which will expand even further north to 63.5 °N by the end of the century due to climate change [24,25]. The most common cultivars are ‘Discovery’, ‘Summerred’, Red Gravenstein, ‘Red Aroma’ and ‘Rubinstep’, while crab apples are mostly used as pollenizers.

Due to the gametophytic self-incompatibility of apples, successful cross-pollination must be accomplished via a cross-compatible pollinator to achieve economically viable yields. The predominant pollinators of apple flowers are considered to be bees (*Apis mellifera*), mason bees (*Osmia* spp.), bumblebees (*Bombus* spp.) and hoverflies, who transfer the pollen grains from the anthers to stigmas [26]. It has been found that yields in apple orchards are positively correlated with insect abundance and the functional diversity of pollinators [27,28]. On the other side, low fruit and seed sets have a negative impact on apple fruit quality, and lead to malformed fruits with low calcium content, which shortens their shelf life [29,30,31]. Furthermore, Garratt et al. [32] have shown that pollination performance accounts for ~65% of market production per hectare, as it affects both the quality and quantity of apples produced. Apple orchards usually have one or two main cultivars and at least two cultivars–pollenizers as pollen donors, with insects as pollen vectors, and it is known from the literature that honeybees have shown a preference for foraging on apple pollen [33]. The aim of this study was therefore to fingerprint the chemical composition of the pollen in Norwegian apple cultivars and try to connect it to its attractiveness to insect pollinators. The results of this study would lead to improving the management of cultivars–pollinizers in commercial apple orchards, which could in return provide higher yields and thus, significant economic benefits. Also, the goal was to determine whether apple pollen could serve as a food and/or food supplement rich in antioxidants.

## 2. Materials and Methods

### 2.1. Plant Material and Pollen Collection

The pollen of 11 different apple cultivars (Table 1) were collected from the intensive orchard in Lofthus, West Norway, from the NIBIO Institute, in the municipality of Ullensvang (latitude 60°19′8.03″ N, longitude 6°39′14.31″ E). The maintenance of the orchard, including fertilization, irrigation and tillage has already been described in Fotirić Akšič et al. [34]. Each cultivar in the orchard is represented by 15 trees, with the exception of ‘Dolgo’ and ‘Professor Sprenger’, which are pole trees. Pollen was gathered in two consecutive years (2022 and 2023). At the balloon stage (code 59), according to the BBCH scale [35], unopened flowers were collected from all the trees and from all the scaffolds around the canopy, transported to the laboratory, and placed in the refrigerator (4 ± 0.5 °C). Unopened anthers were collected in Petri dishes shortly before dehiscence and dried at room temperature for 24 h until pollen shedding started. Afterwards, the closed dish was moved up-and-down and left–right for 2 to 3 min to vibrate the entire dish to increase anther breakage and pollen release. Empty anthers were removed with a dissecting needle. The Petri dishes with the pollen were then kept frozen at −18 °C until chemical analysis [11].

### 2.2. Reagents and Standards and Determination of Sugars and Sugar Alcohols by IC

Sugar standards (glucose, fructose, sucrose, isomaltose, trehalose, sorbitol and mannitol) were purchased from Supelco/Sigma-Aldrich (St. Louis, MO, USA). All the aqueous solutions were prepared using ultrapure water (0.055 µS/cm) obtained by using the Thermo Fisher TKA MicroPure water purification system. A high-performance anion exchange liquid chromatography system with pulsed amperometric detection was used to analyze the sugars and sugar alcohols. A chromatographic measurement was performed using Dionex ICS 3000 DP LC system (Dionex, Sunnyvale, CA 94085, United States) equipped with a quaternary gradient pump and electrochemical detector, which consisted of Au as the working electrode and Ag/AgCl as the reference electrode, autosampler (AS-DV) and Chromeleon software (Chromatography Workstation and Chromeleon 6.7 Chromatography Management Software). All the separations were performed on Carbo Pac PA100 column (4 × 250 mm (analytical) and 4 × 50 mm (guard); Dionex) thermostated to 30 °C. The mobile phase flow rate was 0.7 mL/min, and the mobile phase composition was changed (gradient elution) during the analysis in the following order: −20–5 min = 15% 300 mM NaOH; 5–12 min = 15% 300 mM NaOH and 2% 500 mM NaOAc; 12–20 min = 15% 300 mM NaOH and 4% 500 mM NaOAc; 20–30 min = 20% 300 mM NaOH and 20% 500 mM NaOAc; rest to 100% was ultrapure water. The total analysis run time was 30 min.

### 2.3. Total Phenolic Content Determination

The content of the total phenolics was determined according to the methodology described in the previous publication [36] with slight modification. Namely, the results were expressed as mg/100 g of gallic acid equivalents (GAE) calculated on a fresh weight of sample.

### 2.4. Antioxidant Activity Determination

The antioxidant activity of floral pollen samples collected from different apple cultivars was determined through the application of three different assays: 2,2-diphenyl-1-picrylhydrazyl radical assay (DPPH^⦁^), 2,2′-azino-bis(3-ethylbenzothiazoline-6-sulfonic acid radical cation assay (ABTS^⦁+^) and ferric-reducing power (FRP), based on the methodology previously described [33] with one slight modification. Namely, all the results in the current research were expressed as mg/100 g of Trolox equivalents (TE) calculated on a fresh weight of sample.

### 2.5. Preparation of Pollen Extracts for Determination of Total Phenolic Content, Antioxidative Activity and Chromatographic Analysis

Eleven floral apple pollen (AFP) samples collected from different apple varieties (0.5 g) were extracted with 10 mL 80% methanol containing 0.1%HCl, for 1 h, on a mechanical shaker. After that, the samples were centrifuged at 4000× *g* for 10 min, and the supernatants obtained were further used for the determination of the total phenolic content, antioxidative activity and chromatographic analysis. Immediately prior to UHPLC Q-ToF MS analysis, the samples were filtered through 0.22 µm syringe filters.

### 2.6. UHPLC Q-ToF MS Analysis of Pollen

The analyses were performed on an Agilent 1290 Infinity ultra-high-performance liquid chromatography (UHPLC) system coupled to a quadrupole time-of-flight mass spectrometer (6530C Q-ToF-MS) from Agilent Technologies, Inc., CA, USA, using the same method and operation ESI parameters as previously described in detail by Kostić et al. [37]. The QToF-MS system recorded spectra over the *m*/*z* range from 100 to 1700 in both ionization modes, using the auto MS/MS acquisition mode with collision energy of 30 eV. Agilent MassHunter software was used for instrument control, data evaluation and analysis.

Quantification was performed using the available standards or, in the absence of specific standards, the amounts of each phenolic derivative were quantified using the standards of structurally similar compounds, expressed as a mg/100 g FW pollen sample. The two phenolic standards used for quantification (gentisic acid and quercetin), and their equation parameters, correlation coefficient (r^2^), linear range, LOD and LOQ are shown in Table S1. Accurate masses of components were calculated by using ChemDraw software (version 12.0, CambridgeSoft, Cambridge, MA, USA).

### 2.7. Preparation Extracts for Electrophoretic Analysis

Floral apple pollen protein was extracted according to the procedure described in Kostić et al. [38]. Briefly, the pollen samples were extracted with a phosphate buffer (pH 7.0) at a ratio of 1:10 *w*/*v*, for 1 h, at room temperature, on a mechanical shaker. The samples were than centrifuged at 17,000× *g*, for 15 min. After that, the supernatants were separated, mixed with sample buffers (pH 6.8, contain 5% β-mercaptoethanol) and used for electrophoretic analysis.

#### Sodium Dodecyl Sulfate-Polyacrylamide Gel Electrophoresis (SDS-PAGE)

SDS-PAGE under reducing conditions was performed according to the procedure described in Kostić et al. [38]. In brief, the stacking and separating gels were 12.5% (pH 8.85) and 5% (pH 6.80), respectively. The prepared protein extracts were heated at 90 °C for 5 min and after cooling to room temperature, 25 µL was added to each well. The gels were run in TRIS–glycine buffer solution, pH 8.30, for 3 h. The gels were fixed and stained with 0.23% (*w*/*v*) Coomassie Brilliant Blue R250 for 60 min and destained with 18% (*v*/*v*) ethanol and 8% (*v*/*v*) acetic acid. BlueEasy Prestained Protein Marker (6.5–270 kDa) (Nippon Genetics Europe, Düren, Germany ) was used to estimate the molecular weight of the polypeptides.

SigmaGel software version 1.1 (Jandel Scientific, San Rafael, CA, USA) was used to analyze the scanned gels. The relative content of each identified polypeptide was calculated as a percentage of the corresponding area of the polypeptide relative to the total area of the densitogram.

### 2.8. Raman Instrumentation

The Raman microspectroscopy of the pollen grain samples was recorded using a Horiba Jobin Yvon XploRA, Montpellier, France, Raman spectrometer equipped with an Olympus (Tokyo, Japan) BX 41 microscope. Raman scattering was excited by a laser with a wavelength of 532 nm focused on the sample on the microscope stage through a 50 LWD objective (Olympus, Tokyo, Japan). Raman scattering was performed with 1200 lines/mm grating, resulting in spectra in the range of 100–1800 cm^−1^. The spectral resolution was ~3 cm^−1^ and the calibration was checked using a 520.47 cm^−1^ line of silicon. The spectra were recorded with an exposure time of 10 s after scanning the sample 10 times. Data acquisition and instrument control were performed using LabSpec 6 software (Horiba Scientific, Longjumeau, France). The assignment of the main bands was based on the literature data.

### 2.9. Statistical Analysis

Based on the quantified phenolics, with the aim of gaining a more detailed insight into the data structure and identifying the similarities and specificity of the grouping of objects, Principal Component Analysis (PCA) and hierarchical cluster analysis (HCA) were performed. These analyses were performed in the software package PLS ToolBox, v.6.2.1 MATLAB 7.12.0 (R2011a). All the data were autoscaled before multivariate analysis. PCA and HCA were carried out at the exploratory level, so they were not used as classification models, but rather as hints of what could be expected from the current data and to check if there were some logical patterns in the data that might be explained. PCA for Raman spectroscopy was performed on smoothed, baseline-corrected data normalized to the highest intensity band in the 200 to 1800 cm^−1^ range. The spectra were preprocessed using Spectragryph software (version 1.2.14; SpectroscopyNinja: Oberstdorf, Germany) [39]. The spectra were base-corrected using Savitzky–Golay filters with 7 points and a second-order polynomial function was used for spectrum smoothing. PCA was performed using PAST software (http://palaeo-electronica.org/2001_1/past/issue1_01.htm, accessed on 15 May 2024) [40]. A PC analysis was performed with ten spectra per apple variety/cultivar, resulting in a total of 110 spectra.

A correlation statistical analysis and heat maps were performed based on the content of different classes of phenol compounds—phenolic acid and derivatives (PAD), flavonol aglycones and glycosides (FAG), dihydrochalcone and derivatives (DD) and flavanone and flavan-3-ols (FF) in floral apple pollen samples, combined with the results of TPC, ABTS, DPPH, and FRP tests, in the software package R 4.3.1 software (R Foundation for Statistical Computing, Vienna, Austria; https://www.R-project.org, accessed on 15 May 2024).

## 3. Results and Discussion

### 3.1. Sugars and Sugar Alcohols

In all the apple pollen samples analyzed, five sugars and two sugar alcohols (Table 2) were determined. The sum of the quantified sugar and sugar alcohols varied from 2.52 (‘Elstar’) to 10.17 (‘Summerred’) g/100 g, which corresponded to those obtained by other studies (Table 2) [20,41]. The most dominant sugars in apple floral pollen were fructose, glucose and trehalose, which is not in accordance with Fotirić Akšić et al. [11], who studied the floral pollen of Oblačinska sour cherry clones and found that glucose, fructose and sucrose were the most abundant. This can be attributed to the different species studied and the completely different agro-climatic conditions. In this study, the trehalose content ranged from 0.05 (‘Fryd’) to 4.00 g/100 g (‘Dolgo’), accounting for ~21% of all the quantified sugars on average. As mentioned above, the most abundant sugars in the pollen of the apple cultivars studied were glucose, fructose and trehalose (Figure S1), the only three sugars found in the hemolymph of bees. Trehalose in the hemolymph serves as an indicator of hunger in honeybees and the other insects that use it as an energy store. Trehalose also helps regulate foraging at an individual level, helps with rate crop emptying and helps with foraging decisions [16].

The fructose content ranged from 0.07 (‘Elstar’) to 2.66 g/100 g (‘Discovery’) with an average proportion of ~10%, while the glucose content varied from 0.01 (‘Eden’ and ‘Katja’) to 2.30 g/100 g (‘Discovery’), corresponding to an average porportion of ~6.5%. The level of fructose was much higher than glucose, which is opposite to the findings of Fotirić Akšić et al. [11]. Sucrose-rich pollen was found in the crab apple cultivars ‘Dolgo’ and ‘Professor Sprenger’, but the highest percentage was in the cultivar ‘Aroma’ (9.1%). Sucrose resists the formation of ice crystals in freezing temperatures, preserving pollen viability [9]. The cultivars ‘Discovery’ and ‘Summerred’ were rich in glucose and the cultivars ‘Aroma’, ‘Discovery’ and ‘Summerred’ were rich in fructose. Fructose and glucose are strong phagostimulants for honeybees, although weaker than sucrose [42]. After simple sugars, mannitol was the most abundant sugar alcohol, ranging from 0.32 (‘Elstar’) to 6.22 g/100 g (‘Eden’), accounting for 12.64–89.92% of all the quantified sugars. Moreover, the sum of the quantified sugar alcohols was at the same level as the sugar content in the analyzed pollen samples.

Honeybees prefer sucrose, glucose, fructose, melezitoze, maltose and trehalose when foraging [43]. Carbohydrate-rich pollen provides an alternative energy source, increases colony strength, prevents starvation, and can reduce wintering losses [44].

### 3.2. Total Phenolic Content (TPC)

**Figure 1:**
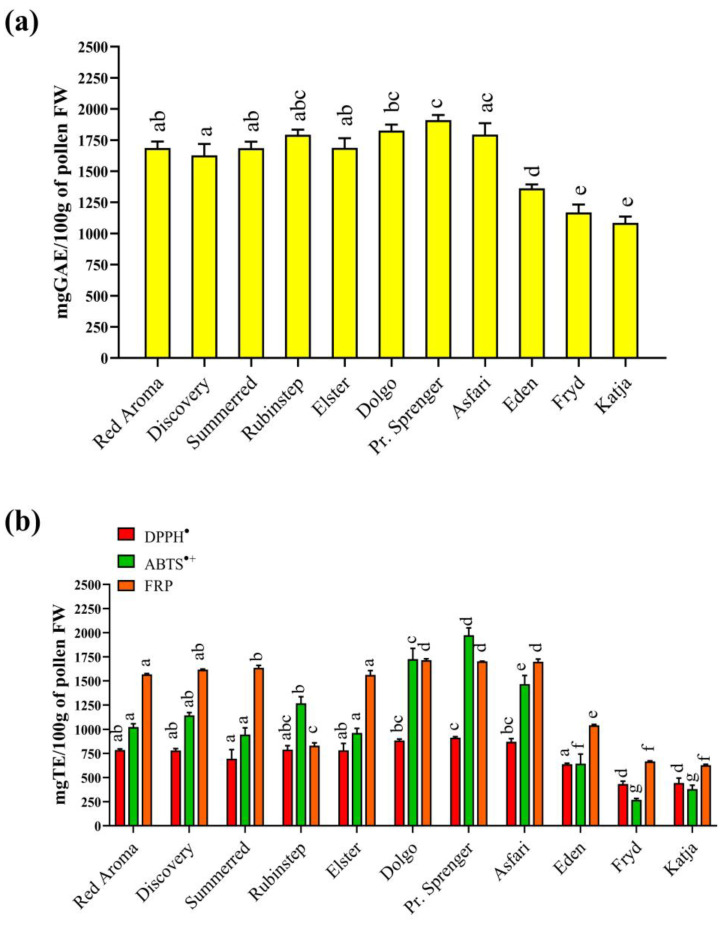
(**a**) Total phenolic content and (**b**) antioxidant properties of different floral apple pollen samples. Values are presented as means ± standard deviations (mean ± SD). Same-colored bars followed by same-lowercase letters are not significantly different (*p* < 0.05), according to Tukey’s test. Abbreviations: TE—Trolox; GAE—gallic acid; ABTS^•+^—ABTS^•+^ scavenging activity; DPPH^•^—DPPH^•^ scavenging activity; FRP—ferric-reducing power.

Phenolic compounds as natural components of the bee diet have been demonstrated to have a positive effect on the longevity of honeybees and their food intake, affect the detoxification capacity of bees [45], attract bee pollinators and enhance their olfactory memory [46]. The determination of TPC is one of the most commonly used methods in pollen analysis to determine the general phytochemical properties of samples. The results obtained are presented in Figure 1a.

In the tested apple pollen samples, the TPC ranged from 1085.1 mg/100 g GAE (‘Katja’) to 1910.7 mg/100 g GAE (‘Professor Sprenger’). Interestingly, the cultivars with the highest TPC were the crab apples ‘Professor Sprenger’ and ‘Dolgo’ (1910.7 and 1827.1 mg/100 g GAE, respectively). This could be related to the origin, as those two cultivars originate from *Malus sylvestris* (“forest apple”) which used to be cultivated in the wild, while the others are cultivars originating from *Malus domestica*. Although the reported results were the highest, they were not significantly different from the following: ‘Asfari’ (1795.0 mg/100 g GAE) and ‘Rubinstep’ (1792.6 mg/100 g GAE). The results obtained were consistent with the results of Moroccan bee-collected samples with a different botanical origin [47]. According to Chaudhary et al. [48], the TPC in *Prunus cerasoides*, *Prunus persica* and *Pyrus pashia* were 14.10, 1.81 and 3.60 mg/g, respectively, while Nozkova et al. [49] reported 0.79 to 1.55 GAE mg/g phenolic contents in *Brassica napus* subsp. *napus* L. However, data about any pollen originating from *Malus* spp. are scarce. The search conducted for this study found only one report of bee-collected pollen samples from Turkey containing apple pollen as an accompanying material [50], which was confirmed via HPLC analysis due to the presence of phloretin and phlorizin-two phenolics, chemotaxonomic markers for the genus *Malus* [51]. However, the authors determined significantly higher TPC values for pollen samples collected in the Ankara and Rize regions (3093.3 and 4137 mg/100 g GAE). The observed differences may be related to the presence of other plant sources that dominate in the pollen samples collected by the bees, to the growing conditions and to the differences in the apple assortment.

### 3.3. Antioxidant Assays

The measurement of antioxidant activity is one of the most common biological activities determined in plant material. There are various assays that have been developed to measure antioxidant activity. In general, the assays can be divided into two major groups: HAT (hydrogen atom transfer)-based methods and SET (single electron transfer)-based methods. HAT-based methods measure the ability for antioxidants to quench free radicals via hydrogen donation, whereas SAT-based methods measure their ability to transfer one electron to reduce any compound (metals, radicals, carbonyls) [52]. Each has its advantages and limitations, which is why it is important to apply several assays in parallel on the same samples [52]. In that sense, antioxidant activity was determined in the current study by applying three different assays: DPPH^⦁^, ABTS^⦁+^ and FRP assays. The mechanisms of these assays differ, as the first two are based on radical quenching ability, while the last one is based on the Fe^3+^ reduction process [52]. Furthermore, a DPPH^⦁^ assay is used for the determination of lipophilic antioxidants, whereas an ABTS^⦁+^ assay is used for the screening of both lipophilic and hydrophilic antioxidants [52]. In this way, we obtained a broader overview of the antioxidant properties of the studied pollen samples. The results obtained are shown in Figure 1b. According to the results for DPPH^⦁^ assay, the range was from 432.8 mg/100 g TE (‘Fryd’) to 914.0 mg/100 g TE, obtained for ‘Professor Sprenger’. The same trend was observed for the other quenching assay, where the ability of the pollen extracts to neutralize free ABTS^⦁+^ was the lowest in the case of the cultivar ‘Fryd’ (269.3 mg/100 g TE), while the highest value was again obtained for the pollen sample of the ‘Professor Sprenger’ cultivar (1973.4 mg/100 g TE). Interestingly, the values determined for the ABTS^⦁+^ assay were significantly higher for several cultivars compared to the DPPH^⦁^ test. It could be assumed that the pollen samples contained a significantly higher amount of hydrophilic antioxidant compounds, as ABTS^⦁+^ mostly detects the activity of these antioxidants, in contrast to DPPH^⦁^, which is recognized as a more lipophilic radical particle [37,52]. The results for the third antioxidant assay applied were significantly lower compared to the previous ones. The range obtained for the FRP assay was from 25.37 mg/100 g TE (‘Fryd’) to 100.6 mg/100 g TE (‘Dolgo’). However, what is consistent in all the assays and also in the TPC is that the ‘wild’ cultivars (‘Dolgo’ and ‘Professor Sprenger’) had the highest values. This could have been provoked by the harsh and unfavorable conditions in the wilderness that cause plants to react more strongly to oxidative stress. Sometimes it is difficult to compare the results for antioxidant assays because the methodology used is different and, also, different standards are used to present the results [52]. However, comparable results for the ABTS^⦁+^ assay were found for the bee-collected pollen samples from Turkey [50] containing *Malus* spp. pollen grains (≈215–237 mg/100 g TE), which had the lowest value obtained for the ‘Fryd’ cultivar. A similar trend was observed in the results of the DPPH^⦁^ assay compared to the results of the same study (≈330 mg/100 g TE) [51], where for all the tested apple cultivars in the current study, the ability to extinguish DPPH^⦁^ quenching was significantly higher than in the results for the bee-collected pollen samples from Turkey containing *Malus* spp. pollen grains [50].

### 3.4. UHPLC Q-ToF MS Analysis of Apple Floral Pollen (AFP)

Various bioactive compounds of apple floral pollen were identified and characterized by UHPLC Q-ToF MS (Table 3), taking into account the exact mass of molecular ions, typical MS fragments and previously published data [5,37,53]. So far, there are only a few studies analyzing polyfloral bee pollen with a proportion of pollen from Rosaceae (mainly the genus *Malus*) [51,53,54] and only one article with scarce characterization of apple (’Quiguan‘ and ‘Gala’) pollen from China [55]. However, this characterization represents a unique fingerprint for apple floral pollen as, to our knowledge; this study is the first time that detailed phytochemical profiles of this pollen have been investigated. In total, four organic acids and sixty-five phenolic compounds were identified in the negative ionization mode and eighteen hydroxycinnamic acid amides (phenylamides) in the positive ionization mode. Due to the wide diversity and for the easier interpretation of the results, all the identified phenolic compounds were categorized into four distinct groups: (I) phenolic acids and derivatives, (II) flavonol aglycones and glycosides, (III) dihydrochalcone and derivatives and (IV) other flavonoids (flavanone and flavan-3-ol aglycone). In addition, all the detected phenolic compounds were also quantified and their amounts were expressed in gentisic acid and quercetin equivalents for phenolic acid and flavonoid derivatives, respectively (Table 4).

Phenolic acids (hydroxycinnamic acid and hydroxybenzoic acid derivatives) were the most abundant class of PCs in AFP, accounting for 37.25% to 63.31% of all the quantified PCs. The majority of the phenolic acids detected were most commonly found in the form of glycosides and esters with quinic acid, and rarely in the form of aglycones (hydroxybenzoic acid and caffeic acid only). Hydroxybenzoic acid derivatives were less abundant than hydroxycinnamic acid derivatives (2.7 to 11.6 times), depending on the apple floral pollen samples collected. The totals of the quantified hydroxybenzoic acids and derivatives and hydroxycinnamic acids and derivatives were the highest in ‘Dolgo’ (211.81 and 794.25 mg/100 g pollen, respectively). Hydroxybenzoic acid hexoside isomer I and both the vanillic acid hexoside isomers were quantified in all the AFP samples and represented the dominant, confirmed hydroxybenzoic acid derivatives. Serra Bonvehí et al. [56] also found vanillic acid and determined that it is a very important constituent of pollen grains that it is responsible for antioxidant activity. Other hydroxybenzoic acid derivatives were selectively detected and quantified in the analyzed pollen samples, such as gallic acid hexoside and dihydroxybenzoic acid hexoside isomer I (quantified only in ‘Dolgo’ and ‘Professor Sprenger’, Table 4). On the other hand, specific vanillic acid derivatives such as vanillin (except ‘Rubinstep’), vanilloside and vanilloloside were only detected, but not quantified (<LOQ). Among the hydroxycinnamic acid derivatives, 3,4-dimethoxycinnamic acid (**13**), coumaric acid hexoside (**14**), caffeic acid hexoside (**19**), and the isomers of coumaroylquinic acid (15 and 16), caffeoylquinic acid (21 and 22) and dicaffeoylquinic acid (23) were detected in significant amounts in all the AFP samples (Table 3 and Table 4). This is in accordance with Almaraz-Abarca et al. [57], who determined that the most common phenolic acids in pollen are chlorogenic, ferulic, cinnamic and caffeic acids. Other derivatives were selectively detected and quantified depending on the apple cultivars from which pollen was collected. For example, ferulic acid hexoside isomer I and diferuloyl hexoside isomer I were found in all the AFP samples, except in the samples ‘Red Aroma’ and ‘Katja’, respectively. Dicaffeoyl hexoside, on the other hand, was only quantified in the sample ‘Fryd’ (12.33 mg/100 g), while it was only present in traces in the other pollen samples. Apple pollen has not been analyzed so far, but these caffeoyl-, feruloyl- and coumaroyl-hexoside and quinic acid derivatives have been detected in various Australian and Serbian apple cultivars [58,59]. Caffeoyldeoxytetronic and sinapic acid hexosides were not quantified (<LOQ), but only detected in some pollen samples.

Among the flavonoids, various flavonol and dihydrochalcone derivatives were most frequently detected (Table 3). All the confirmed flavonol derivatives were glycosides (pentoside, rhamnoside and hexoside derivatives) of kaempferol, quercetin, isorhamnetin and syringetin. However, the content of the individual flavonols detected varied and was strongly dependent on the apple cultivars from which the pollen was collected. The highest levels of flavonol aglycones and glycosides were detected in ‘Katja’ (610.36 mg/100 g) and the lowest in ‘Asfari’ (219.67 mg/100 g). Kaempferol and its two derivatives were detected in all the AFP samples, especially kaempferol-3-*O*-rhamnoside (*m*/*z* 431). The highest content of kaempferol derivatives was found in the samples ‘Rubinstep’ (43.30 mg/100 g) and ‘Eden’ (50.64 mg/100 g), while the content in the other samples was significantly lower. Interestingly, quercetin aglycone was not detected, but its pentosyl, rhamnosyl, and hexosyl derivatives were found at significant levels in all the AFP samples. Compound **56** was recognized as quercetin-3-*O*-(2″-*O*-malonyl)-hexoside, with the typical MS fragments for its identification at 300 *m*/*z*, 463 *m*/*z* and 505 *m*/*z* (loss of CO_2_—44 Da). This compound was quantified only in ‘Dolgo’ and ‘Professor Sprenger’, containing more than 10 mg/100 g, which can used as the marker of the apple species *Malus sylvestris*. The quantification revealed that the total amount of isorhamnetin derivatives was significantly higher than the other flavonol derivatives, with compounds **59**, **61** and **63** being the main contributors. The major fragments used to identify these compounds were at 315 *m*/*z* (Y_0_ – ion) and 314 *m*/*z* (radical anion [Y_0_-H]^−^), which are the typical fragments of the deprotonated isorhamnetin aglycone. In addition, isorhamnetin aglycone and its other derivatives were found in significant amounts only in some of the AFP samples, which can be probably related to the origin of pollen and the apple variety. The total amount and the individual isorhamnetin derivatives (with the exception of compound **62**) were dominantly detected in the pollen samples ‘Eden’ (342.78 mg/100 g), ‘Fryd’ (293.98 mg/100 g) and ‘Katja’ (314.03 mg/100 g). Mentioned and similar quercetin, kaempferol and isorhamnetin glycosides were previously confirmed in various monofloral and polyfloral pollen samples [1,2,3,4,5,6,7,8]. On the other hand, syringetin aglycone and its glycosides were rarely found in pollen. Until now, syringetin aglycone was found only in fermented pollen samples [60], while syringetin-3-*O*-hexoside was confirmed in apple pollen from China [55]. Other syringetin glycosides were reported for the first time in these AFP samples. In addition, syringetin aglycone and its hexoside were also previously detected in some apple cultivars [61] possibly justifying the presence of these derivatives in apple pollen. Compound **65** (*m*/*z* 507) was identified as syringetin-3-*O*-hexoside, with major fragments appearing at [345/344 *m*/*z* (Y_0_^−^/[Y_0_-H]^−^)→330/329 *m*/*z* (-CH_3_, −15Da)→314 *m*/*z* (-CH_3_, −15Da)]. In addition, compounds **67** (*m*/*z* 653) and **68** (*m*/*z* 669) were recognized as syringetin-3-*O*-(2″-*O*-rhamnosyl)-hexoside and syringetin-3-*O*-(2″-*O*-hexosyl)-hexoside, respectively. The most important fragments for their identification were Y_0_^−^ (345 *m*/*z*) and the radical anion ([Y_0_–H]^−^) (344 *m*/*z*), which are characteristic of the deprotonated syringetin aglycone. In addition, both the compounds had a fragment at 489 *m*/*z* resulting from the loss of hexosyl moiety + H_2_O ([M − H-146-18]^−^) in compound **67** or rhamnosyl moiety + H_2_O ([M − H-162-18]^−^) for compound **68**, indicating a 1→2 interglycosidic linkage between the sugar units. The identified compounds **69** and **70** are structurally more complex syringetin derivatives than the previously mentioned compounds (**67** and **68**), as they additionally contain a malonyl moiety in their structure, as indicated by the fragments at 695 *m*/*z* (compound **69**) and 711 *m*/*z* (compound **70**) (-CO_2_, 44Da). The syringetin aglycone was dominantly quantified in all the AFP samples (10.27–73.95 mg/100 g), followed by its derivatives **65** and **67** (Table 3), while the other syringetin derivatives were selectively quantified and detected in the pollen samples. For example, the compound detected as syringetin-3-*O*-(6″-acetyl)-hexoside was only detected in significant amounts in the samples ‘Elstar’, ‘Fryd’ and ‘Katja’.

Dyhydrochalcones, primarily phloretin (*m*/*z* 273) and 3-hydroxyphloretin (*m*/*z* 289) derivatives, represent a special group of phenolic compounds that were detected in the analyzed apple pollen samples. It is well known that phloretin and its 2′-glycoside (phlorizin) are among the most abundant phenolic compounds in different apple cultivars [9,10]. Moreover, Bayram et al. [51] proposed phlorizin as a potential chemotaxonomic marker for *Malus* pollen. Compounds **75** (*m*/*z* 567) and 80 (*m*/*z* 583), were identified as phloretin- and 3-hydroxyphloretin-2′-*O*-(6″-pentosyl)-hexosde, respectively. The key fragments for their identifications were 273 *m*/*z* (deprotonated phloretin) and [289 *m*/*z* (deprotonated 3-hydroxyphloretin)→271 *m*/*z* (-H_2_O, −18 Da)], obtained via losses of the pentosyl-hexoside moiety ([M − H-294 Da]). However, special attention should be paid to the different acylated phloretin derivatives detected in these AFP samples (compounds **73**, **74**, **76**, **77** and **78**, Table 3). These compounds have the same MS fragments at 273 *m*/*z* and 167 *m*/*z*, but different monoisotopic masses and formulas. Phloretin-4′-*O*-(6″-coumaroyl)-hexoside (compound **77**) and phloretin-4′-*O*-(6″-feruloyl)-hexoside (compounds **78**) were previously confirmed in crab apple leaves and proposed as potential anticancer agents [62]. These compounds (**77** and **78**), along phloretin aglycone and phlorizin, were detected and quantified in all the AFP samples (Table 3 and Table 4). To our knowledge, other acylated phloretin derivatives (benzoyl, cinnamoyl, and caffeoyl) have not been identified in apple fruits and pollen, so far (Table 3). The highest total amount of dihydrochalcone was detected in the ‘Professor Sprenger’ (380.27 mg/100 g) and ‘Discovery’ (267.08 mg/100 g) samples (Table 3).

**Figure 2:**
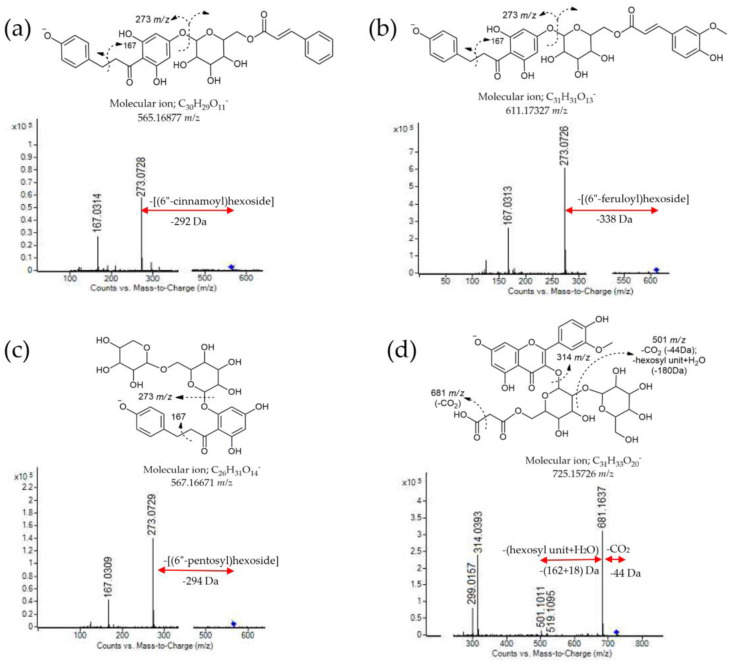
MS fragmentation patterns and proposed structures with major fragments: (**a**) phloretin-4′-*O*-(6″-cinnamoyl)-hexoside; (**b**) phloretin-4′-*O*-(6″-feruloyl)-hexoside; (**c**) phloretin-2′-O-(6″-pentosyl)-hexoside; (**d**) isorhamnetin-3-*O*-(2″-hexosyl-6″-malonyl)-hexoside, (ESI^−^, CE = 30 eV).

Naringenin and epicatechin belong to the group of other flavonoids that were detected in the AFP samples. However, they were mostly found in small amounts, except in sample ‘Professor Sprenger’, in which the epicatechin content was 43.52 mg/100 g. Finally, the total amount of all the quantified phenolic compounds in the apple pollen samples ranged from 1079.45 mg/100 g (‘Red Aroma’) to 1525.63 mg/100 g (‘Professor Sprenger’) (Table 3). In addition to the phenolic compounds, several typical organic acids, such as malic acid, quinic acid and citric acid, as well as ispropylmalic acid derivatives, were also detected in the AFP samples (Table 3). Characteristic MS/MS fragmentation patterns and the proposed structure of some phenolics compounds (compounds **63**, **74**, **75** and **78**, Table 3) are shown in Figure 2.

In recent years, bee-collected pollen has been recognized as an excellent source of various hydroxycinnamic acid amides (phenylamides) [2,5,6,11], which show pronounced biological activity [63]. In contrast, the phenylamides of floral pollen have hardly been investigated. A total of eighteen different and well-known putrescine and spermidine derivatives were identified in the analyzed AFP samples (compounds **32**–**49**, Table 3). Spermine derivatives were not detected. Most of the identified phenylamides contained one or more coumaroyl moieties, or less frequently caffeoyl, feruloyl and acetyl residues. Looking at the relative content (Table S2), the total spermidine derivatives (53.94–65.64%), were slightly more represented in the AFP samples, than in the total putrescine derivatives (34.36–46.06%). Dicoumaroyl putrescine was the most abundant putrescine derivative with a relative content higher than 15% of the total phenylamides, followed by coumaroyl putrescine isomer II and coumaroyl caffeoyl putrescine. In contrast, among the spermidine derivatives, tricoumaroyl and dicoumaroyl spermidine dominated, with a share of more than 40% of the total detected phenylamides. The relative contents of the other detected phenylamides were significantly lower.

### 3.5. Floral Apple Pollen Protein Composition–Electrophoretic Analysis

Pollen is the most desirable and attracting source of protein for honeybees, which also has an impact on pollinator visits. Protein is important for brood rearing, overall colony development, and the longevity of adult workers, so it can be stated that the nutritional value of protein is the most important factor in the selection of pollen as food for honeybees [64]. According to Pernal and Currie [65], honeybees do not necessarily prefer flowers with higher protein values. On the contrary, bumblebees visit flowering plant species with a higher pollen protein content [66] and collect pollen with higher protein content than honeybees [67].

**Figure 3:**
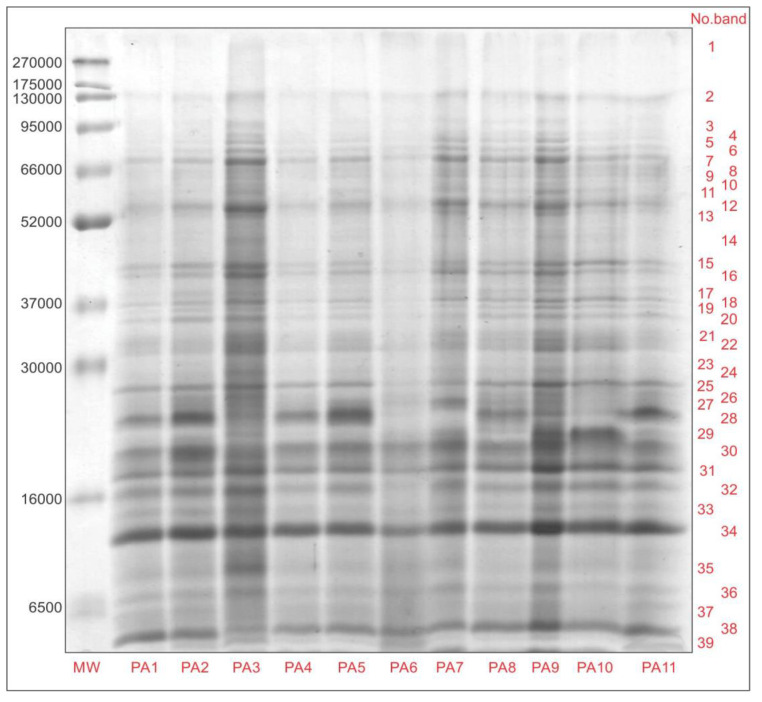

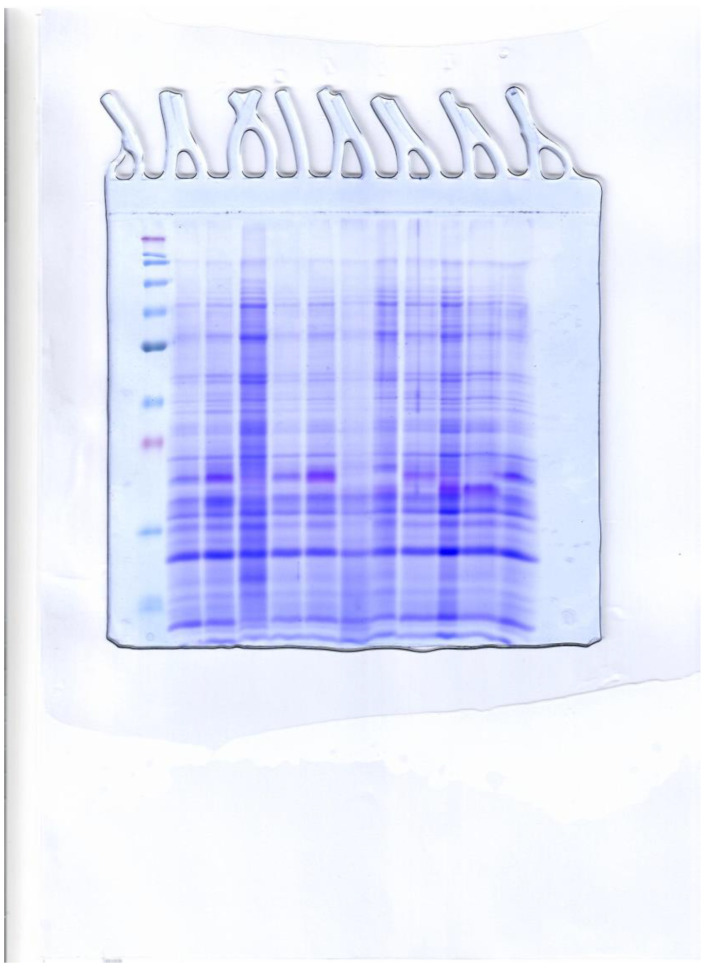
SDS-PAGE patterns of extractable proteins of different floral apple pollen samples. Abbreviations: PA1—Red Aroma; PA2—Discovery; PA3—Summerred; PA4—Rubinstep; PA5—Elstar; PA6—Dolgo; PA7—Professor Sprenger; PA8—Asfari; PA9—Eden; PA10—Fryd; PA11—Katja; MW—molecular weight standards.

The protein extracts of the flowering apple pollen were separated on SDS-PAGE under reducing conditions into a large number of polypeptides (39 bands) with a MW ranging from 300 kDa to less than 6.5 kDa (Figure 3). Similar results were obtained for the protein profile of the floral pollen protein extracts of allergenic plants in the Philippines [68]. These results differ from those reported for bee-collected pollen where a significantly lower number of protein bands were observed [16,17]. A lower number of protein bands in bee-collected floral pollen was also reported by Gupta [69] for *Helianthus annuus*. Considering that the majority of the proteins detected in the pollen samples were proteins involved in metabolic processes and biosynthesis in plants [69], a reduction in the amount of some of these proteins was expected after processing by bees. It has been reported that hand-collected pollen (floral pollen) contains three to four times higher amounts of most proteins compared to bee-collected pollen [70].

Significant differences between the protein profiles of the different apple pollen samples can be observed with regard to the number and relative content of the proteins (Table 5) as well as their intensity (Figure 3). All the identified protein bands can be divided into eight protein ranges, of which 30–16 kDa and 6.5–16 kDa ranges dominate with 30.58–39.74% and 17.55–26.55% of the extractable proteins, respectively. The lowest relative content of the polypeptides with a molecular weight higher than 95 kDa was found, but those were the most abundant in ‘Dolgo’ and ‘Professor Sprenger’. All the pollen samples contained polypeptides in the 6.5–16 kDa range, while the number of the polypeptides in the other protein ranges varied among the apple pollen samples. The greatest differences were in the protein ranges of 16–30 kDa and 52–66 kDa. The pollen from the cultivars ‘Summerred’ and ‘Eden’ were the richest in different proteins with the highest intensity in the SDS-PAGE gel, while ‘Red Aroma’ and ‘Rubinstep’ apple pollen contained the lowest number of different proteins. On the other hand, the lowest intensity of protein bands on the SDS-PAGE gel was observed for ‘Dolgo’ apple pollen, indicating the lowest extractable protein content.

### 3.6. Raman Spectral Fingerprinting of Apples Pollen Grains

**Figure 4:**
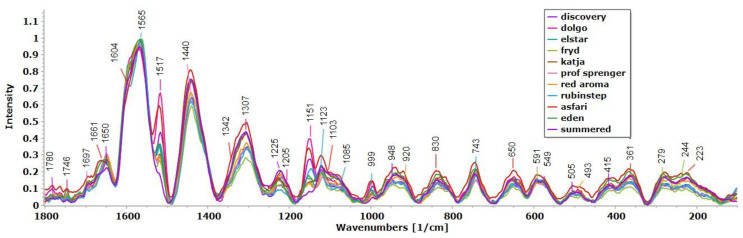
Averages of normalized Raman spectra of eleven apple cultivar pollen samples, recorded in the spectral range from 100 to 1800 cm^−1^, with bands specific for phenolic compounds 1606, ~1570 and 1205, 830 cm^−1^), carotenoids (999, 1151, and 1517 cm^−1^), lipids (~1746, 1440, 1307 cm^−1^), proteins (549, ~1660 cm^−1^) and glycosidic structure (~940, 1123, 1103, 1085, and specific bands below 990 cm^−1^).

The average Raman spectra of the pollen samples in the so-called fingerprint region (100–1800 cm^−1^) are shown in Figure 4, while the characteristic bands and the corresponding assignments are listed in Table 6. The obtained Raman spectra of the pollen grains contained information about the main chemical constituents of pollen, such as lipids, proteins and carbohydrates, as well as the biopolymers of the pollen grain wall, sporopollenins and cellulose.

Phenolic compounds are components of pollen that contribute to its antioxidant properties [37]. The most striking features are the signals associated with phenylpropanoids at ~1565 cm^−1^ in the average Raman spectra (Figure 4), which are correlated with phenyl ring vibrations, indicating the presence of sporopollenin with its shoulder at 1604 cm^−1^, thus marking the exine of the pollen grain, which is probably associated with cinnamic and p-coumaric acids as important precursors of sporopollenins [71,72]. The second band with a higher intensity at 1440 cm^−1^ indicates the CH_2_/CH_3_ deformation vibrations in lipids, while the band with medium intensity at 1307 cm^−1^ [73,74] or the last band could indicate proteins [75]. The bands related to sporopollenin compounds, the most abundant metabolites in pollen [73], appeared with a higher intensity in the range of 1560–1630 cm^−1^ [71,76,77], together with bands in the range of 830–890 cm^−1^ [72].

Although carotenoid pigment was present in a low concentration in the pollen, the carotenoid-associated Raman bands followed the unique spectral pattern of the carotenoids: at about 1517 cm^−1^ (medium intensity), 1151 cm^−1^ (medium intensity) and 999 cm^–1^ (lower intensity), the stretching of the C=C (ν1), C−C (ν2) bonds and the in-plane vibrations of the C-CH_3_ group, respectively [72].

The interior of the pollen grains had a complex composition of proteins, lipids and especially carbohydrates [73]. The spectral range was between 1630 and 1680 cm^−1^ where the amide I and II bands of the proteins were typically located. The band at 1659 cm^−1^ could be assigned to the amide II and C=O stretching vibrations, which are normally representative of proteins [73,74,78]. A band at 549 cm^−1^ was discovered in amide II [79].

It is known that starch, whose two naturally occurring components are amylose and amylopectin, forms granules in the vegetative cell of pollen [80]. The literature data indicate that the structural components of starch have bands in the range between 920 and 1130 cm^−1^, such as 920, 948, 1085, 1103 and 1123 cm^−1^ [80]. The spectra of the pollen tube are dominated by the bands in the 1000–1200 cm^−1^ range of cellulose (Figure 3), e.g., 1123 cm^−1^ [80]. The abundance of pectin and cellulose can be explained by the composition of the outer wall of the pollen tube and the inner pollen grain layer (intine), which are known to consist of cellulose, hemicelluloses and pectin. The preliminary assignments of the bands recorded in the pollen samples (Figure 4; Table 6) at wavelengths below 540 cm^−1^ could be assigned to mono-, di- and polysaccharides corresponding to C−O−C, C−C and C−O stretching vibrations [75,79].

**Table 6:** Vibrational bands and their assignments in apple pollen cultivars average spectra and literature data.

Recorded Bands	Literature Data	Vibrational Mode	Chemical Moiety	Reference
1746	1750	CH_2_, C=O	Lipids	[73,74]
1661	1660, 1662, 1669	C=O	Amide I	[73,75,78]
1650	1650, 1655, 1660, 1640, 1630	C=O	Amide I	[71,72,73,74,75,78,79]
1604	~1600, ~1610	Phenyl C=C ring vibrations	Sporopollenin (cinnamic and p-coumaric acid), Phe, Tyr	[72,73,74,75,78,79]
1565	~1570	Phenyl C=C ring vibrations	Sporopollenin	[71,72]
1517	1519	C=C	Carotenoid	[71,72,75]
1440	1440	CH2/CH3 def. of aliphatic carbon chains	Lipids	[71,73,74,75]
1342	/	Non-identifed	/	/
1307	1304	N–H, CH_2_	Lipids amide III	[73,74,75,79]
1225	1228	Phosphate, C-O aryl vibration		[75,79]
1205	1209	Arom ring str	Sporopollenin	[72,73,74]
1151	1152	C−C	Carotenoid	[71,72,76]
1123, 1103	1123, 1097	C–O–H	Amylose, cellulose	[79]
1085	1085	C–O–C, C–O–H	Amylose, starch	[73,79]
999	1000	C−CH_3_ in-plane group rocking vibrations	Carotenoid	[71,72,76]
948	940, 949	C–O–C, C–OH	Starch	[72,73,78,79]
920	920	C–O–C, C–OH	Starch	[72,73]
830	820–860 833	C–O–C, C–C	Sporopollenins Phenylpropanoid acids	[72,73,75]
743	/	Non-identified	/	/
650	/	Non-identified	/	/
591	590	Arom ring def	Sporopollenin, Phe	[75]
549	549	C=O	Amide II	[78,79,81]
505, 493		C-O-C, C-C-O, C-C-C	Starch, pectin	[75,81]
415		C-O-C, C-C-O, C-C-C	Carbohydrate	[75]
361		C-O-C, C-C-O, C-C-C	Carbohydrate	[75]
279		C-O-C, C-C-O, C-C-C	Carbohydrate	[75]
244		C-O-C, C-C-O, C-C-C	Carbohydrate	[75]
223		C-O-C, C-C-O, C-C-C	Carbohydrate	[75]

The pre-processed spectra of all the pollen grains indicated a very similar chemical composition between the cultivars (Figure 4). According to the intensity of the averaged spectra of the apple pollen grains, the cultivar ‘Asfari’ was to some extent richer in saturated aliphatic components from lipids (higher intensity at 1440 cm^−1^), followed by ‘Fryd’, ‘Eden’ and ‘Rubinstep’, which also had higher intensities of aromatic components from sporopolenins (bands around 1600 cm^−1^), while ‘Asfari’, ‘Dolgo’ and ‘Rubinstep’ were richer in carotenoids (corresponding to bands at 1517, 1151 and 999 cm^−1^). In addition, ‘Asfari’ and ‘Eden’ had higher carbohydrate contents than the other cultivars.

### 3.7. Multivariate Analysis

Principal Component Analysis based on the content of the phenolic compounds in eleven different apple pollen samples led to a model with seven components that explained 89.41% of the total variability of the data. The statistical parameters (number of principal components and the percentage of variance explained by them) were shown in Table S3.

**Figure 5:**
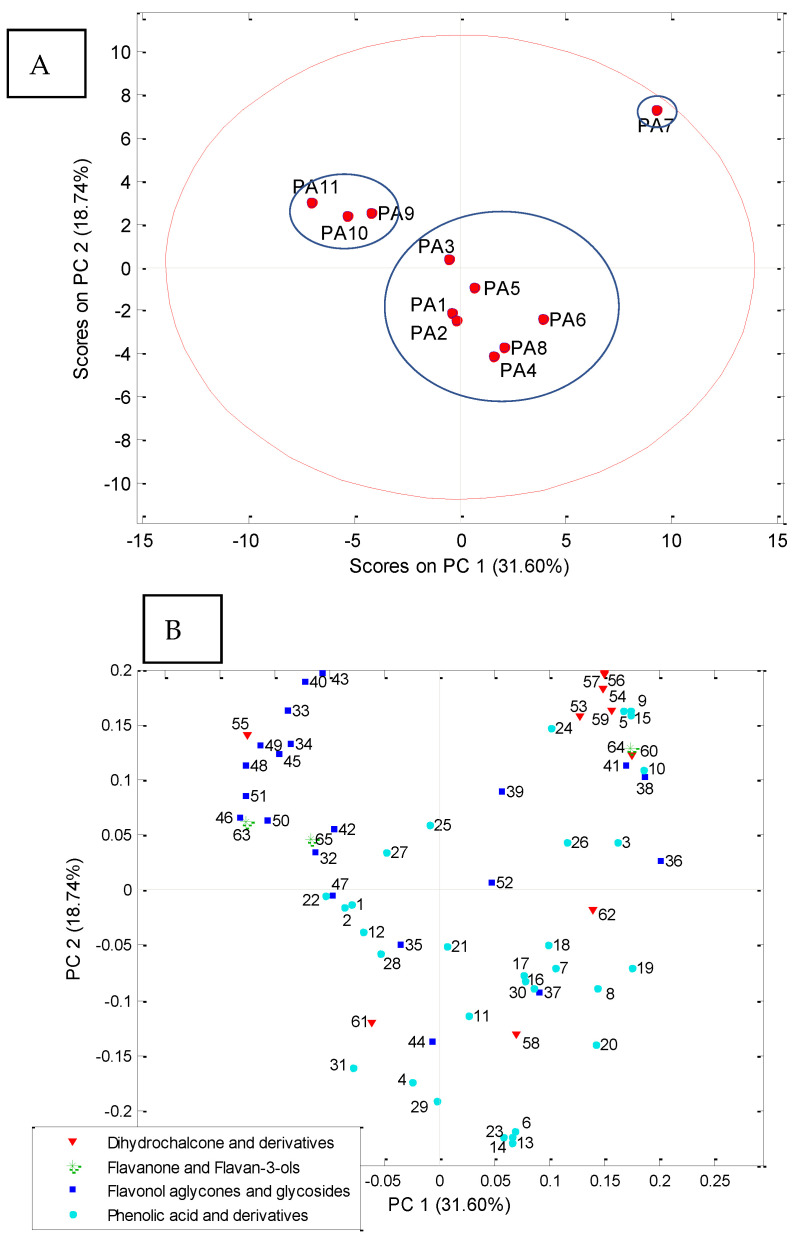
PCA based on the content of the polyphenolic compounds in eleven different floral apple pollen samples: (**A**)—score plot and (**B**)—loading plot. The designations (serial numbers: 1–65) of the individual compounds belonging to the shown classes of polyphenols on the loading plot are consistent with the designations in Table 4. PA1—‘Red Aroma’; PA2—‘Discovery’; PA3—‘Summerred’; PA4—‘Rubinstep’; PA5—‘Elstar’; PA6—‘Dolgo’; PA7—‘Professor Sprenger’; PA8—‘Asfari’; PA9—‘Eden’; PA10—‘Fryd’; PA11—‘Katja’.

The mutual projections of the factor scores and their loadings for the first two PCs, which explained 50.34% of the total variance, are shown in Figure 5. The results were obtained based on the content of the polyphenolic compounds in different apple floral pollen samples (Table 4).

Three groups of objects stand out in the graph of scores. The first consists of the sample ‘Professor Sprenger’, the second encompasses the samples ‘Eden’, ‘Fryd’ and ‘Katja’, while the samples ‘Red Aroma’, ‘Discovery’, ‘Summerred’, ‘Rubinstep’, ‘Elstar’, ‘Dolgo’ and ‘Asfari’, fall into the third group (Figure 5A). Dihydrochalcone and derivatives, whose concentrations are the highest in the pollen of ‘Professor Sprenger’ (367.95 mg/100 g) have the greatest positive influence on the separation of this sample along the PC1 axis (Figure 5B, Table 4). Of the total ten quantified dihydrochalcones and derivatives, phloretin, 3-hydroxyphloretin, phloretin-4′-*O*-(6″-benzoyl)-hexoside, phloretin-4′-*O*-(6″-cinnamoyl)-hexoside, phloretin-4′-*O*-(6″-caffeoyl)-hexosid and phloretin-4′-*O*-(6″-coumaroyl)-hexoside are those that have the highest concentrations in this sample and therefore influence the separation from the other samples (Figure 5B, Table 4). The negative influence along the PC1 axis was mainly due to flavonol aglycones and glycosides, namely kaempferol, isorhamnetin-3-*O*-(2″-*O*-hexosyl)-hexoside, and syringetin-3-*O*-(2″-*O*-rhamnosyl)-hexoside, which were present in the lowest concentrations in the pollen of ‘Professor Sprenger’. This was followed by syringetin-3-*O*-hexoside, quercetin-3-*O*-pentoside, quercetin-3-*O*-hexoside, which was the most abundant, and syringetin-3-*O*-(6″-*O*-acetyl)-hexoside, syringetin-3-*O*-(2″-*O*-hexosyl)-hexoside, isorhamnetin-3-*O*-(2″-rhamnosyl-malonyl)-hexosid and syringetin-3-*O*-(2″-rhamnosyl-malonyl)-hexoside which were not quantified in this pollen sample (Figure 5B, Table 4).

In the separation of the pollen ‘Eden’, ‘Fryd’ and ‘Katja’, the flavonol aglycones and glycosides had the greatest positive influence along the PC2 axis: isorhamnetin, syringetin, syringetin-3-*O*-hexoside, syringetin-3-*O*-(6″-*O*-acetyl)-hexoside, isorhamnetin-3-*O*-(2″-*O*-malonyl)-hexoside, isorhamnetin-3-*O*-(2″-*O*-rhamnosyl)-hexoside, isorhamnetin-3-*O*-(2″-*O*-hexosyl)-hexoside, syringetin-3-*O*-(2″-*O*-hexosyl)-hexoside, isorhamnetin-3-*O*-(2″-hexosyl-malonyl)-hexoside and syringetin-3-*O*-(2″-rhamnosyl-malonyl)-hexoside, whose concentrations were highest in these samples, and isorhamnetin-3-*O*-(2″-rhamnosyl-malonyl)-hexoside, which was only quantified in these samples. Quercetin-3-*O*-pentoside was present in lower concentrations in these pollen samples, while quercetin 3-*O*-(2″-*O*-malonyl)-hexoside was not quantified. (Figure 5, Table 4). Besides the flavonol aglycones and glycosides, the most positive influence along the PC2 axis on the separation of these samples from the others and flavanones and flavan-3-ols were, namely, naringenin and (epi)catechin-hexoside, which were present in the highest concentrations in these samples, while the others were quantified from the classes of flavanones and flavan-3-ols—epicatechin was responsible for the differentiation of the pollen from ‘Professor Sprenger’ because it was the most abundant in that sample (Figure 5, Table 4). It should also be noted that the highest concentrations of phlorizin from the class of dihydrochalcone and derivatives were recorded in the samples of the second group (‘Eden’, ‘Fryd’ and ‘Katja’) (Figure 5, Table 4).

The third group consisted of pollen samples from ‘Red Aroma’, ‘Discovery’, ‘Summerred’, ‘Rubinstep’, ‘Elstar’, ‘Dolgo’ and ‘Asfari’. Their separation was mainly influenced by phenolic acid and derivatives, with ‘Dolgo’ pollen having the highest concentration of these compounds—813.60 mg/100 g pollen (Figure 5, Table 4). The separation was also affected by dihydrochalcones and derivatives such as phloretin-2′-*O*-(6″pentosyl)-hexoside, 3-hydroxyphloretin-2′-*O*-(6″-pentosyl)-hexoside, and phloretin-4′-*O*-(6″-feruloyl)-hexoside, which were present in most of the samples of the third group at higher concentrations than in other samples. It should be noted that the differentiation of the ‘Dolgo’ pollen from the others was influenced by the presence of kaempferol-3-*O*-rhamnoside and quercetin 3-*O*-rhamnoside, which belong to the class of flavonol aglycones and glycosides. They were the most abundant in this sample compared to all the other samples, while kaempferol-3-*O*-(2″-caffeoyl)-pentoside had the lowest concentration in this sample, apart from the fact that it was not quantified in the pollen of ‘Professor Sprenger’.

**Figure 6:**
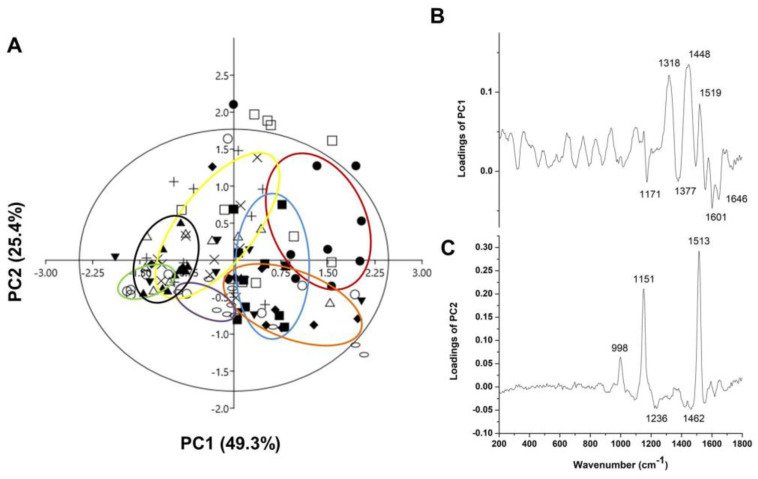
PCA applied to the data obtained from Raman spectra of pollen apple cultivars: (**A**) score plot, (**B**,**C**) loading plots; closed cycle—‘Asfari’; open cycle—‘Professor Springer’; closed square—‘Eden’; open square—‘Dolgo’; closed triangle—‘Fryd’; triangle—‘Elstar’; inverted closed triangle—‘Red Aroma’; diamond—‘Katja’; plus—‘Discovery’; oval—‘Summerred’; ×—‘Rubinstep’.

A multivariate analysis based on PCA was performed to analyze the Raman spectra of the pollen samples of 11 apple cultivars. Figure 6 shows the results and loadings of PCA1 versus PC2. Figure 6A shows the breakdown into different object groups, with the first and second principal components describing 74.7% of the data variance. The PCA of the Raman data shows that the predominant spectral differences were the result of variations in bands associated with proteins, sporopollenin derivatives, carotenoids and lipids (Figure 6). Multivariate methods were used to integrate the different spectroscopic data sets and to extract and visualize the common underlying patterns of the mutual information of the different spectroscopic data to enable the interpretation of the spectroscopic measurements. For this purpose, the loadings for each PC were analyzed to determine which bands had the greatest influence so that it could be determined which major chemical constituent was represented by multiple bands in the Raman spectra. The score plot of PC1 against PC2 (Figure 6A) shows a separation between the pollen of the apple cultivars ‘Eden’, Katja’, ‘Asfari’ from ‘Professor Sprenger’, ‘Summerred’ and ‘Fryd’. According to PC1, the pollen of ‘Rubinstep’ shares a similar chemical composition to these two groups. The loading plot of the PC1 (Figure 6B) showed the loadings responsible for the previously mentioned separation of the pollen ‘Eden’, ‘Katja, and ‘Asfari’ from ‘Professor Sprenger’, ‘Dolgo’, ‘Summerred’ and ‘Fryd’.

PC1 typically explains most of the variability; therefore, the bands in the spectral range of 1000–1650 cm^−1^ were mainly assigned to proteins and phenolic compounds. The PC1 loading plots showed high factor loadings associated with the amide I group (negative loadings) at 1646 cm^−1^ and cinnamic and *p*-coumaric acids according to the negative loadings at 1601 and 1171 cm^–1^, and could originate from the water-soluble part of proteins according to 1377 cm^−1^ [78]. Loadings making positive contributions were mainly assigned to CH_2_/CH_3_ deformation vibrations in lipids (1448 cm^−1^), sinapic acid (1318 cm^−1^), phenylpropanoid acids (831 cm^−1^), and carotenoids (999, 1152 and 1519 cm^–1^) [71,75,76]. According to the analyzed loadings of PC1, ‘Summerred’, ‘Fryd’, ‘Dolgo’ and ‘Professor Sprenger’ had higher contents of proteins and sporopolenins than ‘Asfari’, ‘Eden’ and ‘Katja’ and they were richer in lipids and carotenoids than ‘Summerred’, ‘Fryd’ and ‘Professor Sprenger’. Both sporopollenins and carotenoids probably play roles in protecting pollen grains under various abiotic stress conditions. The exine was predominantly composed of sporopollenins, a chemically resistant and extremely robust biopolymer, while the exine was covered in a sticky, lipid-rich pollen coat that contained carotenoids, which play a role in protecting against oxidative stress and also serve as an attractor for pollinators.

The loading plot of PC2 (Figure 6C) shows the bands responsible for the separation between the pollen of ‘Professor Sprenger’, ‘Dolgo’, ‘Summerred’ and ‘Katja’ from the pollen of ‘Asfari’ and ‘Rubinstep’, corresponding to the negative loadings originating predominantly from lipids (e.g., 1236 and 1462 cm^−1^) [75]. The bands with positive loadings indicating the carotenoids, such as 998, 1151 and 1513 cm^−1^, were also responsible for the differentiation [71,72,76]). According to the PC2 negative loadings (1236 and 1462 cm^−1^), the pollen of ‘Professor Sprenger’ and ‘Summerred’ had lower concentrations of lipids and carotenoids than the pollen of ‘Asfari’ and ‘Rubinstep’.

### 3.8. Correlation Analysis

**Figure 7:**
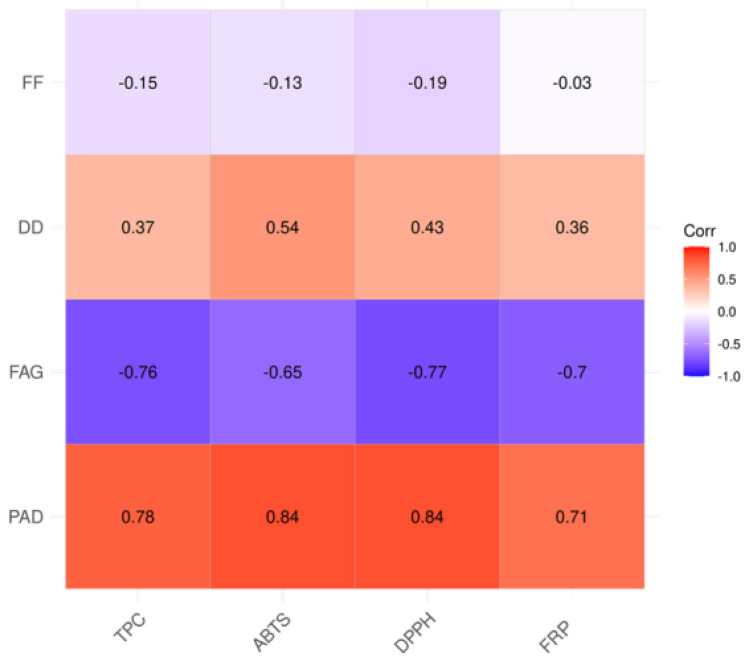
Heat map based on the content of different classes of phenol compounds—phenolic acid and derivatives (PAD), flavonol aglycones and glycosides (FAG), dihydrochalcone and derivatives (DD) and flavanone and flavan-3-ols (FF) in floral apple pollen samples, combined with the results of TPC, ABTS^•+^, DPPH^•^ and FRP tests.

A heat map is a data visualization that uses a color-coding scheme to display various values and it is used in various forms of analytics [82]. In this work, they were used to facilitate the monitoring of the results obtained and to determine the possible correlation and linkage between the content of the polyphenol compounds and the results of spectrophotometric analysis and antioxidant properties in different apple floral pollen samples (Figure 7). The results obtained show that the most positive correlation exists between the values of total phenolic content and the antioxidant properties of the different apple floral pollen samples and quantified phenolic acid and derivatives (PAD) (Table 4). A positive correlation was also found between the results for TPC, ABTS^•+^, DPPH^•^, and FRP and dihydrochalcone and its derivatives (Table 4). A positive correlation was also observed between the results for TPC, ABTS^•+^, DPPH^•^ and FRP and dihydrochalcone and its derivatives (Table 4). This result was quite expected, as it was already well-documented that in floral pollen the most important and the strongest antioxidants are indeed phenolic acids/derivatives [83,84], as they protect the pollen grains from destructive UV-B light, which can impair their functionality. This was observed in *Vicia faba*, *Helleborus foetidus*, and *Betula pendula* pollens [83]. In the context of the lower positive correlation of dihydrochalcone/derivatives and antioxidant activity, it can be hypothesized that the glycosylation of the OH group on phloretin aglycone reduced the antioxidant activity of the derivates, as suggested in the literature [84]. In the case of quantified flavanone and flavan-3-ols (FF) and flavonol aglycones and glycosides (FAG) (Table 4) and total phenolic content and antioxidant properties, the results showed a negative correlation. Flavonol aglycones and glycosides (FAG) had the most negative influence on the values for total phenolic content and antioxidant properties.

## 4. Conclusions

To our knowledge, this is the first comprehensive chemical analysis of apple (*Malus* sp.) floral pollen. The findings presented in this study revealed that the composition of apple pollen varies greatly due to different phylogenetic origins. The crab apples ‘Dolgo’ and ‘Professor Sprenger’ exhibited unique pollen chemical profiles by having the highest content of sucrose, TPC, DPPH, ABTS, FRP, hydroxybenzoic acids and its derivatives, quercetin and its derivatives, dihyrochalcone, epicatechin and putrescine derivates, and proteins with a molecular weight 66–95 kDa and >95 kDa. Only the pollen of these genotypes contained quercetin-3-*O*-(2″-*O*-malonyl)-hexoside, which can be used as a marker for apple species *Malus sylvestris*. Because of all these properties, especially the polyphenols that prevent oxidative damages in insects, honeybees and other insects are showing preference for these particular genotypes, making them the best pollen suppliers for apple cultivars. On the other hand, the commercial cultivar ‘Summerred’ was characterized by the highest sugar content; the pollen of the cultivars ‘Summerred’ and ‘Eden’ were the richest in various proteins, while the pollen of ‘Rubinstep’ had the highest content of carotenoids, and ‘Asfari’ of lipids. All this leads to a “win-win” situation, as the combination of crab apples with economically important cultivars in the same orchard provides a balanced diet for pollinators from the insects’ point of view and promotes their health, while giving producers higher yields.

In addition to the practical application of these results in fruit production, the chemical content of apple pollen has shown that it is an excellent source of bioactive compounds that can have a positive impact on human health by being used as an apitherapeutic product, for the prevention and/or cure of diseases (cancer, diabetes, cardiovascular diseases and arteriosclerosis) or from the nutritional point of view, by being incorporated into diet and enriching it with phenolics, proteins and carotenoids, thus becoming a “superfood”. 

## Figures and Tables

**Table 1:** Apple cultivars used in this study.

Cultivar	Species	Parents	Country of Origin
‘Red Aroma’	*Malus domestica*	‘Ingrid Marie’ × ‘Filippa’	Denmark
‘Discovery’	*Malus domestica*	‘Worcester Pearmain’ × ‘Beauty of Bath’	England
‘Summerred’	*Malus domestica*	Open pollinated ‘Summerland’ (‘McIntosh’ × ‘Golden Delicious’)	Canada
‘Rubinstep’	*Malus domestica*	‘Clivia’ × ‘Rubin’	Czech Republic
‘Elstar’	*Malus domestica*	‘Golden Delicious’ × ‘Ingrid Marie’	The Netherlands
‘Dolgo’	*Malus sylvestris*		Russia
‘Professor Sprenger’	*Malus sylvestris*		The Netherlands
‘Asfari’	*Malus domestica*	‘Elstar’ × ‘Delcorf Apache’	Belgium
‘Eden’	*Malus domestica*	‘Magic Star^®^’ × ‘Honeycrisp’	Belgium
‘Fryd’	*Malus domestica*	‘Magic Star^®^’ × ‘Honeycrisp’	Belgium
‘Katja’	*Malus domestica*	‘James Grieve’ × ‘Worcester Pearmain’	Sweden

**Table 2:** Contents of sugars and sugar alcohols in analyzed pollen samples (g/100 g).

Cultivar	Sorbitol	Mannitol	Trehalose	Glucose	Fructose	Sucrose	Isomaltose	Sum
Red Aroma	0.130 ± 0.003 bc*	1.73 ± 0.03 c	1.42 ± 0.06 b	0.99 ± 0.04 b	1.93 ± 0.21 b	0.66 ± 0.04 b	0.35 ± 0.03 c	7.21 c
Discovery	0.251 ± 0.005 d	1.86 ± 0.03 c	1.34 ± 0.06 b	2.3 ± 0.1 c	2.66 ± 0.29 c	0.11 ± 0.01 a	0.29 ± 0.02 bc	8.81 c
Summerred	0.285 ± 0.006 d	2.73 ± 0.04 d	2.70 ± 0.12 c	1.39 ± 0.06 b	1.31 ± 0.14 b	0.62 ± 0.04 b	1.13 ± 0.09 d	10.17 d
Rubinstep	0.096 ± 0.002 b	1.09 ± 0.02 b	1.21 ± 0.05 b	0.109 ± 0.005 a	0.36 ± 0.04 a	0.25 ± 0.02 a	0.37 ± 0.03	3.49 a
Elstar	0.146 ± 0.003 c	0.32 ± 0.01 a	1.38 ± 0.06 b	0.20 ± 0.01 a	0.07 ± 0.01 a	0.24 ± 0.02 a	0.17 ± 0.01 b	2.52 a
Dolgo	0.59 ± 0.02 e	3.18 ± 0.05	4.00 ± 0.18 d	0.069 ± 0.003 a	0.74 ± 0.08 a	1.10 ± 0.07 c	0.27 ± 0.02 b	9.95
Professor Sprenger	0.186 ± 0.004 c	1.64 ± 0.03 c	1.65 ± 0.07	0.21 ± 0.01 a	0.34 ± 0.04 a	1.20 ± 0.08 c	0.01 ± 0.01 a	5.23 b
Asfari	0.050 ± 0.001 a	4.56 ± 0.07 e	0.27 ± 0.01 a	0.053 ± 0.002 a	0.09 ± 0.01 a	0.21 ± 0.01 a	-	5.22 b
Eden	0.099 ± 0.002 b	6.2 ± 0.1 f	0.16 ± 0.01 a	0.013 ± 0.001 a	0.23 ± 0.03 a	0.20 ± 0.01 a	-	6.92 b
Fryd	0.052 ± 0.001 a	4.45 ± 0.07 e	0.048 ± 0.002 a	0.019 ± 0.001 a	0.23 ± 0.03 a	0.16 ± 0.01 a	-	4.95 b
Katja	0.059 ± 0.001 a	5.04 ± 0.08 e	0.070 ± 0.003 a	0.011 ± 0.001 a	0.30 ± 0.03 a	0.16 ± 0.01 a	-	5.65 b

* Different letters within the same column indicate statistically significant differences at *p* < 0.05 via Tukey’s test.

**Table 3:** Characterization and identification of bioactive compounds in different floral apple pollen samples, using UHPLC Q-ToF MS. Target compounds, mean expected retention times (RT), molecular formula, calculated mass, *m*/*z* exact mass, mean mass accuracy (mDa), and MS fragments are presented.

RT	Compound Name	Formula, [M − H]^−^ or [M + H]^+^	Calculated Mass, [M − H]^−^ or [M + H]^+^	*m/z* Exact Mass, [M − H]^−^ or [M + H]^+^	mDa	MS Fragments (%)	No
***Phenolic acids and derivatives***							
***Hydroxybenzoic acid and derivatives***							
5.66	**Hydroxybenzoic acid**	C_7_H_5_O_3_^−^	137.02390	137.02191	1.99	**/**	1
1.62	**Hydroxybenzoic acid hexoside isomer I**	C_13_H_15_O_8_^−^	299.07670	299.07628	0.42	**137.02116(100)**, 138.0252(14)	2
4.50	**Hydroxybenzoic acid hexoside isomer II**	C_13_H_15_O_8_^−^	299.07670	299.07405	2.65	136.0128(30), **137.02074(100)**, 138.02368(10)	3
1.95	**Dihydroxybenzoic acid hexoside isomer I**	C_13_H_15_O_9_^−^	315.07160	315.07314	−1.54	**108.01851(100)**, 109.02572(41), 110.02841(3), 133.02813(1), **152.00799(60)**, 153.01395(15)	4
3.23	**Dihydroxybenzoic acid hexoside isomer II**	C_13_H_15_O_9_^−^	315.07160	315.06941	2.19	**108.01872(100)**, 109.02564(38), 110.02918(3), **152.00791(62)**, 153.01392(14), 154.01766(2)	5
4.85	**Vanillin**	C_8_H_7_O_3_^−^	151.03950	151.03830	1.20	103.92313(13), 105.03101(75), 108.04371(10), 120.01837(28), **121.02538(100)**, 122.02788(15)	6
5.32	**Vanilloside**	C_14_H_17_O_8_^−^	313.09230	313.08923	3.07	106.03891(4), 107.0459(20), 109.0255(4), 113.02149(2), **123.04161(100)**, 124.04358(5), 151.03722(5)	7
5.12	**Vanilloloside**	C_14_H_19_O_8_^−^	315.10800	315.10207	5.93	109.02563(4), **123.04141(100)**, 124.04557(11), 128.03565(1), 153.05192(59), 154.0536(7)	8
3.47	**Vanillic acid hexoside isomer I**	C_14_H_17_O_9_^−^	329.08730	329.08485	2.45	**108.01847(100)**, 109.02204(8), **123.04155(36)**, 124.04527(4), **152.00786(67)**, 153.01165(8), **167.03119(33)**,	9
4.45	**Vanillic acid hexoside isomer II**	C_14_H_17_O_9_^−^	329.08730	329.08498	2.32	**108.01864(100)**, 109.02243(8), **123.04176(37)**, 124.04518(4), **152.0081(68)**, 153.0117(7), **167.03159(36)**	10
2.22	**Gallic acid hexoside**	C_13_H_15_O_10_^−^	331.06650	331.06554	0.96	107.0113(2), 123.00641(4), 124.01288(23), **125.02125(97)**, 126.02506(7), **168.0027(100)**, 169.00788(14)	11
5.52	**Syringic acid hexoside**	C_15_H_19_O_10_^−^	359.09780	359.09490	2.90	123.00566(37), **138.02879(100)**, 139.03227(9), 153.05223(25), 154.05575(3), 166.99446(11), 182.01819(60), 183.02227(6), **197.04192(27)**, 198.04382(4)	12
***Hydroxycinnamic acid and derivatives***							
8.76	**3,4-Dimethoxycinnamic acid**	C_11_H_11_O_4_^−^	207.06570	207.06290	2.80	101.03693(6), **103.05236(100)**, 104.05574(10), 119.04685(56), 120.05079(6), **147.04141(31)**, 165.05222(3)	13
6.68	**Coumaric acid hexoside**	C_15_H_17_O_8_^−^	325.09230	325.08949	2.81	117.03144(22), 118.03506(2), **119.04679(6)**, **145.02593(100)**, 146.02915(11), **163.03694(2)**	14
6.40	**Coumaroylquinic acid isomer I**	C_16_H_17_O_8_^−^	337.09230	337.08784	4.46	111.04205(4), **119.04701(100)**, 155.03198(3), **163.03639(51)**, 164.03972(6), 173.04267(2), **191.05224(7)**	15
7.21	**Coumaroylquinic acid isomer II**	C_16_H_17_O_8_^−^	337.09230	337.08986	2.44	111.04184(18), **119.04713(49)**, 137.02099(12), 155.03155(6), **163.03658(26)**, **173.04219(100)**, 174.0449(9), 175.04662(2), **191.05288(7)**	16
6.87	**Caffeic acid**	C_9_H_7_O_4_^−^	179.03440	179.03437	0.03	106.04154(6), 107.04559(6), 108.02205(5), 109.02439(4), 134.03373(75), **135.04108(100)**, 136.04699(6)	17
7.68	**Caffeoyl deoxytetronic acid**	C_13_H_13_O_7_^−^	281.06610	281.06345	2.65	105.0161(12), 117.03216(8), **134.03342(100)**, 135.03681(12), 149.05736(4), 178.03037(4)	18
6.00	**Caffeic acid hexoside**	C_15_H_17_O_9_^−^	341.08730	341.08551	1.79	133.02604(22), 134.03124(4), 135.04162(19), **161.02098(100)**, 162.02436(11), **179.03079(9)**	19
8.35	**Dicaffeoyl hexoside**	C_24_H_23_O_12_^−^	503.11900	503.12129	−2.29	135.04202(25), 161.02077(30), **179.03107(100)**, 180.03425(11), 221.04139(3), 300.02205(5), 301.03057(2), 323.07278(15), 324.07569(3)	20
5.73	**Caffeoylquinic acid isomer I**	C_16_H_17_O_9_^−^	353.08730	353.08445	2.85	134.03412(5), **135.04199(88)**, 136.04529(9), 155.03159(2), 161.02054(5), 173.04158(3), 179.03125(42), **191.05265(100)**, 192.05574(10)	21
6.61	**Caffeoylquinic acid isomer II**	C_16_H_17_O_9_^−^	353.08730	353.08451	2.79	127.03663(2), 135.04119(1), 161.02043(2), 173.04193(2), **191.05223(100)**, 192.05582(9)	22
8.56	**Dicaffeoylquinic acid**	C_25_H_23_O_12_^−^	515.11900	515.11622	2.78	135.04174(16), 161.02063(5), 173.0418(4), **179.03168(63)**, **191.05216(100)**, 192.05617(8), **353.08368(11)**	23
9.03	**Coumaroyl-caffeoylquinic acid**	C_25_H_23_O_11_^−^	499.12400	499.12251	1.49	119.04674(9), 135.04166(17), 145.02654(9), 161.02038(6), **163.03645(48)**, 173.04165(11), 175.03513(5), **179.03127(43)**, **191.05217(100)**, **337.08971(3)**, **353.08228(4)**	24
6.47	**Ferulic acid hexoside isomer I**	C_16_H_19_O_9_^−^	355.10290	355.09474	8.16	**134.03356(100)**, 135.03813(12), 149.05719(24), 178.02328(15), 179.03053(2), **193.04861(13)**	25
7.41	**Ferulic acid hexoside isomer II**	C_16_H_19_O_9_^−^	355.10290	355.09961	3.29	**134.03392(100)**, 135.03837(12), 149.05709(28), 160.01304(60), 161.0172(9), 175.03636(95), 176.04314(48), 177.03876(8), 178.02431(14), 191.06759(38), **193.04678(48)**	26
9.10	**Diferuloyl hexoside isomer I**	C_26_H_27_O_12_^−^	531.15030	531.14386	6.44	134.03391(14), 135.03868(2), 149.05752(12), 160.01295(5), 161.02253(2), 175.03658(31), 176.04043(4), 178.02341(18), 179.02894(3), **193.04684(100)**, 323.07166(2), 337.08948(9), 338.09245(2)	27
9.90	**Diferuloyl hexoside isomer II**	C_26_H_27_O_12_^−^	531.15030	531.14808	2.22	134.03405(7), 149.05745(3), 160.01295(21), 161.01814(3), **175.0366(100)**, 176.04024(14), 191.06746(4), **193.04672(24)**, 217.04731(3), 235.0577(8), 265.06772(2), 295.07836(4), 337.0884(7)	28
7.48	**Feruloylquinic acid**	C_17_H_19_O_9_^−^	367.10290	367.10101	1.89	111.0419(12), 127.03717(2), 129.0161(2), 134.03391(30), 135.0374(3), 149.05819(2), 155.03209(2), 173.04201(11), 175.03699(1), **191.05266(100)**, 192.0559(9), **193.04771(17)**	29
9.30	**Feruloylcaffeic acid hexoside**	C_25_H_25_O_12_^−^	517.13460	517.13113	3.47	133.02547(6), 135.0417(6), **161.02084(100)**, 162.02429(12), 175.03659(3), **179.03089(13)**, **193.04684(5)**, 235.0566(5), 295.07891(2)	30
6.74	**Sinapic acid hexoside**	C_17_H_21_O_10_^−^	385.11350	385.11055	2.95	113.02213(7), 119.03317(4), 121.02567(3), 149.02057(42), 150.02304(7), 151.04031(3), 163.10578(3), **164.04393(100)**, 165.04518(8), 179.06841(22), 191.05192(6), **208.0342(22)**, **223.05432(10)**	31
***Hydroxycinnamic acid amides***							
***Putrescin derivatives***							
4.20	**Coumaroyl putrescine isomer I**	C_13_H_19_N_2_O_2_^+^	235.14410	235.14503	−0.92	**119.05034(100)**, 120.05438(12), **147.04476(95)**	32
6.00	**Coumaroyl putrescine isomer II**	C_13_H_19_N_2_O_2_^+^	235.14410	235.14532	−1.21	**119.04990(75)**, 120.05383(8), **147.04466(100)**	33
6.74	**Acetyl coumaroyl putrescine**	C_15_H_21_N_2_O_3_^+^	277.15520	277.16500	−9.80	114.10386(5), 119.05061(18), 120.0543(2), **147.04512(100)**, 148.04836(12)	34
9.23	**Dicoumaroyl putrescine**	C_22_H_25_N_2_O_4_^+^	381.18140	381.18389	−2.49	119.04955(13), **147.0447(100)**, 148.04765(16), 218.11796(9), 219.12087(2), 235.14509(6)	35
5.32	**Caffeoyl putrescine**	C_13_H_19_N_2_O_3_^+^	251.13902	251.13988	−0.86	107.05201(7), 117.03487(37), 135.04526(56), 145.02945(48), 162.07814(9), **163.03968(100)**	36
8.76	**Coumaroyl caffeoyl putrescine**	C_22_H_25_N_2_O_5_^+^	397.17630	397.17849	−2.19	119.05016(8), 135.04464(4), 145.0289(7), **147.04442(100)**, 148.04842(12), **163.03924(61)**, 164.04344(7), 218.11843(11), 219.12137(2), 234.11333(5), 235.14418(8), 251.13959(4)	37
8.36	**Dicaffeoyl putrescine**	C_22_H_25_N_2_O_6_^+^	413.17130	413.17670	−5.40	135.04424(5), 145.02901(8), **163.03898(100)**, 234.11269(11), 235.11576(2), 251.13936(9)	38
6.46	**Feruloyl putrescine**	C_14_H_21_N_2_O_3_^+^	265.15520	265.15412	1.08	117.03507(39), 118.03845(5), 134.03801(3), **145.02995(100)**, 146.03301(13), 149.06094(18), **177.05582(45)**	39
9.36	**Coumaroyl feruloyl putrescine**	C_23_H_27_N_2_O_5_^+^	411.19202	411.19859	−6.57	117.03461(6), 119.05011(3), 145.02894(37), 147.04492(47), **177.05538(100)**, 235.14457(5)	40
***Spermidine derivatives***							
3.44	**Coumaroyl spermidine**	C_16_H_26_N_3_O_2_^+^	292.20250	292.20324	−0.74	112.11279(8), 119.05003(16), **147.04462(100)**, 148.04781(14), 149.04978(1), 204.10353(2), 218.11811(3)	41
9.97	**Dicoumaroyl spermidine isomer II**	C_25_H_32_N_3_O_4_^+^	438.23930	438.24128	−1.98	119.05011(6), **147.04523(100)**, 148.04879(11), **204.10333(74)**, 205.10621(12), 218.11903(12), 275.17792(15), 292.20385(39), 293.20666(9), 421.21704(5), 438.24302(5)	42
7.95	**Dicoumaroyl spermidine isomer I**	C_25_H_32_N_3_O_4_^+^	438.23930	438.24134	−2.04	119.05028(6), 129.13953(3), **147.04475(97)**, 148.0479(14), **204.10266(100)**, 205.10542(22), 218.11761(14), 275.17648(14), 292.20248(36), 293.20543(10), 438.24036(8)	43
10.11	**Tricoumaroyl spermidine**	C_34_H_38_N_3_O_6_^+^	584.27611	584.27928	−3.17	**147.04502(46)**, **204.10302(53)**, 275.17729(18), 292.20361(27), 420.23035(30), **438.24163(100)**	44
7.61	**Coumaroyl caffeoyl spermidine**	C_25_H_32_N_3_O_5_^+^	454.23420	454.23685	−2.65	**147.04493(63)**, **163.03962(50)**, **204.10246(100)**, 205.10556(16), 218.11826(7), **220.09769(54)**, 221.10796(10), 234.11315(9), 275.17611(11), 291.17134(8), 292.2018(29), 293.20503(7), 308.19744(24)	45
9.76	**Dicoumaroyl caffeoyl spermidine**	C_34_H_38_N_3_O_7_^+^	600.27100	600.27508	−4.08	**147.0447(37)**, **163.03957(5)**, **204.10243(46)**, 205.10623(7), 275.17727(15), 292.20325(18), 293.20571(5), 308.19857(10), 438.23795(13), 439.24249(4), **454.23543(100)**, 455.23842(40)	46
8.15	**Diferuloyl spermidine**	C_27_H_36_N_3_O_6_^+^	498.26040	498.26193	−1.53	145.02967(16), **177.05612(100)**, 178.05999(12), 207.06546(5), **234.11363(78)**, 235.11806(18), 305.19163(9), 322.21536(41), 323.21729(10), 481.24186(4), 498.26193(18), 499.26601(8)	47
8.07	**Coumaroyl feruloyl spermidine**	C_26_H_34_N_3_O_5_^+^	468.24980	468.25212	−2.32	145.0298(15), **147.04575(30)**, **177.05568(91)**, 178.05933(11), 204.10358(7), 218.11911(11), **234.11367(100)**, 235.1184(17), 275.17829(4), 292.20441(31), 293.2073(8), 322.2144(11), 468.25237(12)	48
10.18	**Dicoumaroyl feruloyl spermidine**	C_35_H_40_N_3_O_7_^+^	614.28660	614.29093	−4.33	**147.04457(28)**, **177.05516(20)**, 204.10272(37), 205.10568(6), 275.17654(18), 292.2026(15), 322.21375(9), 438.24022(16), 439.24275(6), 450.24011(27), 451.24069(13), **468.25028(100)**, 469.25447(39)	49
***Flavonol aglycones and glycosides***							
***Kaempferol and derivatives***							
10.65	**Kaempferol**	C_15_H_9_O_6_^−^	285.04046	285.03807	2.39	137.01806(14), 143.04616(12), 159.04274(13), 169.06395(11), 171.04227(11), 227.02816(13), 229.04565(12), **285.03685(100)**	50
9.03	**Kaempferol-3-*O*-rhamnoside**	C_21_H_19_O_10_^−^	431.09780	431.09486	2.94	167.03136(12), 227.03074(17), 228.03391(3), 229.04647(7), 255.02551(35), 256.03171(12), 257.04112(8), **284.02828(100)**, 285.03481(98), 431.09522(3)	51
10.58	**Kaempferol-3-*O*-(2″-caffeoyl)-pentoside**	C_29_H_23_O_13_^−^	579.11390	579.11117	2.73	119.04697(15), 135.04203(7), 161.02031(23), 167.03081(16), 179.03112(21), 273.07266(42), 284.02787(14), **285.03621(100)**	52
***Quercetin and derivatives***							
8.29	**Quercetin-3-*O*-pentoside**	C_20_H_17_O_11_^−^	433.07710	433.07418	2.92	151.00039(5), 178.99513(3), 243.026(2), 255.02592(9), 271.02113(17), **300.02387(100)**, 301.0298(44)	53
8.56	**Quercetin-3-*O*-rhamnoside**	C_21_H_19_O_11_^−^	447.09329	447.09113	2.15	151.00018(8), 178.99427(6), 243.02576(2), 255.02571(9), 271.02108(14), **300.02357(100)**, 301.03023(70), 302.03304(13), 447.09113(2)	54
8.09	**Quercetin-3-*O*-hexoside**	C_21_H_19_O_12_^−^	463.08770	463.08461	3.09	151.00027(4), 178.99675(3), 243.02612(2), 255.02545(6), 271.02096(12), **300.02391(100)**, 301.02983(45), 302.03285(8), 463.08461(2)	55
8.36	**Quercetin-3-*O*-(2″-*O*-malonyl)-hexoside**	C_24_H_21_O_15_^−^	549.08800	549.09078	−2.78	150.9999(2), 178.99483(1), 255.02579(3), 271.02161(5), 272.02413(2), **300.02326(100)**, 301.0289(44), 302.03184(8), 353.08461(1), 371.20109(1), 463.0827(4), 505.0947(10)	56
***Isorhamnetin and derivatives***							
10.65	**Isorhamnetin**	C_16_H_11_O_7_^−^	315.05050	315.04771	2.79	109.99779(58), 137.99242(22), 165.98716(50), 216.03889(28), 227.03061(21), 229.01063(19), 243.02535(25), 255.02559(32), 256.03198(15), 271.02064(21), **300.02336(100)**, 301.02656(21)	57
8.43	**Isorhamnetin-3-*O*-hexoside**	C_22_H_21_O_12_^−^	477.10330	477.10221	1.09	243.026(1), 255.02565(4), 271.02134(19), 272.026(6), **299.01593(100)**, 300.02225(50), 301.02528(9), **314.03923(46)**, 315.04484(19), 316.04814(4), 477.09914(5)	58
8.70	**Isorhamnetin-3-*O*-(2″-*O*-malonyl)hexoside**	C_25_H_23_O_15_^−^	563.10370	563.10414	−0.44	255.02482(5), 271.02042(9), 272.02707(5), 299.01571(69), **300.0228(86)**, 301.02615(16), **314.0392(100)**, 315.04585(75), 519.10947(12), 520.11297(4)	59
7.95	**Isorhamnetin-3-*O*-(2″-*O*-rhamnosyl)-hexoside**	C_28_H_31_O_16_^−^	623.16176	623.16146	0.30	271.02022(5), 299.01578(44), 300.02102(13), **314.03929(100)**, 315.04402(30), 459.09002(2)	60
7.75	**Isorhamnetin-3-*O*-(2″-*O*-hexosyl)-hexoside**	C_28_H_31_O_17_^−^	639.15610	639.15522	0.88	271.02061(6), 299.01575(46), 300.02139(20), 301.02526(4), **314.03876(100)**, 315.04437(45), 459.08813(5), 639.15169(57), 640.15602(23)	61
8.29	**Isorhamnetin-3-*O*-(2″-rhamnosyl-6″-malonyl)-hexoside**	C_31_H_33_O_19_^−^	709.16160	709.15973	1.87	245.08653(16), 299.01595(22), 300.02294(7), **314.03893(100)**, 315.04304(41), **477.09566(34)**, 478.09593(10), 503.11065(6), 665.16689(80), 666.16824(39)	62
8.02	**Isorhamnetin-3-*O*-(2″-hexosyl-6″-malonyl)-hexoside**	C_31_H_33_O_20_^−^	725.15650	725.15726	−0.76	271.02093(3), 299.01569(25), 300.02244(10), **314.0393(72)**, 315.04471(30), 501.10108(4), 519.10951(2), **681.16378(100)**, 682.16645(41)	63
***Syringetin and derivatives***							
10.78	**Syringetin**	C_17_H_13_O_8_^−^	345.06159	345.05680	4.79	109.99756(17), 138.99983(12), 149.02112(19), 164.97943(32), 243.02591(23), 259.02077(16), 271.02049(17), 287.01597(19), 315.01085(78), 316.01337(16), **330.03454(100)**, 331.03685(21)	64
8.55	**Syringetin-3-*O*-hexoside**	C_23_H_23_O_13_^−^	507.11390	507.11478	−0.88	286.00826(7), 301.03162(16), 302.03497(3), 314.00272(2), **329.02655(100)**, 330.03089(27), 331.03453(5), **344.04981(29)**, 345.05397(10), 507.11066(12), 508.11203(4)	65
8.83	**Syringetin-3-*O*-(6″-*O*-acetyl)-hexoside**	C_25_H_25_O_14_^−^	549.12440	549.12488	−0.48	286.00936(5), 287.01499(6), 301.02914(7), 302.03518(11), 314.0083(3), **329.02504(100)**, 330.03298(67), 331.03721(13), **344.04855(74)**, **345.05743(37)**, 549.12063(6)	66
8.02	**Syringetin-3-*O*-(2″-*O*-rhamnosyl)-hexoside**	C_29_H_33_O_17_^−^	653.17180	653.17166	0.14	286.0089(2), 301.032(6), 314.00922(2), 329.02603(51), 330.03023(17), 331.03492(3), **344.04923(100)**, 345.05443(32), 346.05553(6), 489.10589(2), 653.16742(50), 654.17165(20)	67
7.82	**Syringetin-3-*O*-(2″-*O*-hexosyl)-hexoside**	C_29_H_33_O_18_^−^	669.16670	669.16846	−1.76	301.03153(7), 329.02541(60), 330.03067(29), 331.03945(5), **344.04997(100)**, 345.05497(52), 489.09743(4), 669.16273(81), 670.16648(29)	68
8.35	**Syringetin-3-*O*-(2″-rhamnosyl-6″-malonyl)-hexoside**	C_32_H_35_O_20_^−^	739.17220	739.17622	−4.02	274.06715(2), 329.02476(32), 330.03149(11), 331.02472(2), 343.04504(2), **344.05001(77)**, 345.05516(23), 507.10638(2), 531.10621(3), **695.17793(100)**, 696.18165(41), 697.18544(13)	69
7.61	**Syringetin-3-*O*-(2″-hexosyl-6″-malonyl)-hexoside**	C_32_H_35_O_21_^−^	755.16710	755.17041	−3.31	329.02716(10), 343.04169(25), 344.0484(12), **345.05686(59)**, 346.05923(13), 387.068(17), 506.10208(40), 507.10867(42), **549.12052(100)**, 550.12257(31), 591.12989(24), 711.17232(82), 712.17547(37)	70
***Dihydrochalcone and derivatives***							
10.51	**Phloretin**	C_15_H_13_O_5_^−^	273.07630	273.07336	2.94	119.04713(77), 120.05022(8), **123.04207(100)**, 124.0449(10), 125.02151(27), 149.0205(4), 151.0001(18), 166.02379(3), **167.03139(47)**, 168.03575(5), 179.03097(3), 189.05214(20)	71
8.96	**Phlorizin**	C_21_H_23_O_10_^−^	435.12910	435.12522	3.88	119.04741(4), 123.04197(11), 125.02092(11), 149.02096(1), **167.03131(100)**, 168.0345(10), 179.03118(11), **273.07283(55)**, 274.0765(12)	72
11.46	**Phloretin-4′-*O*-(6″-benzoyl)-hexoside**	C_28_H_27_O_11_^−^	539.15530	539.15135	3.95	123.04213(4), 167.03152(56), 168.03385(6), **273.07201(100)**, 274.07703(21)	73
11.72	**Phloretin-4′-*O*-(6″-cinnamoyl)-hexoside**	C_30_H_29_O_11_^−^	565.17100	565.16877	2.23	123.04197(5), 167.03137(47), 168.03374(5), **273.07284(100)**, 274.07638(18)	74
8.49	**Phloretin-2′-*O*-(6″-pentosyl)-hexoside**	C_26_H_31_O_14_^−^	567.17146	567.16671	4.74	123.04146(3), 125.02106(5), 167.03093(28), **273.07291(100)**	75
10.11	**Phloretin-4′-*O*-(6″-caffeoyl)-hexoside**	C_30_H_29_O_13_^−^	597.16136	597.15946	1.90	125.01969(29), **135.04115(25)**, 161.02086(20), 167.03113(68), **273.07301(100)**, **435.07586(18)**	76
10.51	**Phloretin-4′-*O*-(6″-coumaroyl)-hexoside**	C_30_H_29_O_12_^−^	581.16645	581.16153	4.92	167.03146(38), **273.07312(100)**	77
10.65	**Phloretin-4′-*O*-(6″-feruloyl)-hexoside**	C_31_H_31_O_13_^−^	611.17702	611.17327	3.75	167.03134(33), 191.03157(5), 209.04169(4), **273.07264(100)**, 297.07234(10), 315.08316(5)	78
9.70	**3-Hydroxyphloretin**	C_15_H_13_O_6_^−^	289.07120	289.06787	3.33	109.02616(7), **123.04138(100)**, 124.04481(10), 125.02078(39), 135.041(15), 150.99938(9), 161.05586(5), 167.0315(74), 168.03428(7), 179.03461(3), 187.03663(1), 188.03959(2)	79
8.49	**3-Hydroxyphloretin-2′-*O*-hexoside**	C_21_H_23_O_11_^−^	451.12400	451.12041	3.59	109.02592(2), 123.04229(9), 125.02098(22), **167.03115(100)**, 168.03488(10), 271.05668(4), 272.06111(1), **289.06746(14)**, 290.07015(2)	80
8.09	**3-Hydroxyphloretin-2′-*O*-(6″-pentosyl)-hexoside**	C_26_H_31_O_15_^−^	583.16630	583.16519	1.11	123.04405(2), 125.02095(29), **167.03115(96)**, 168.03423(8), 245.08069(11), 246.08852(7), 247.09512(6), 271.05776(10), **289.06728(100)**, 290.07137(22)	81
***Flavanone and Flavan-3-ols***							
10.51	**Naringenin**	C_15_H_11_O_5_^−^	271.06060	271.05721	3.39	107.01109(15), **119.04705(100)**, 120.05044(9), 123.04165(98), 124.0445(6), 125.02133(29), 135.04151(3), 151.00024(27), 152.00319(4), 167.0309(46), 189.05195(20)	82
7.28	**Epicatechin**	C_15_H_13_O_6_^−^	289.07120	289.06865	2.55	109.02633(99), 121.02579(27), 122.03394(20), **123.04149(100)**, 125.02054(46), 135.03957(13), 137.01982(20), 149.02079(20), 151.0349(40), 161.05708(16), 187.03869(12), 203.0676(23), 221.07693(14)	83
***Organic acid and derivatives***							
0.74	**Malic acid**	C_4_H_5_O_5_^−^	133.01370	133.01134	2.36	107.03461(5), **115.00018(100)**, 117.0057(3), 133.01289(5), 134.04167(3)	84
5.99	**Isopropylmalic acid**	C_7_H_11_O_5_^−^	175.06060	175.05724	3.36	113.05706(54), 114.06148(5), **115.03661(100)**, 116.04104(7), 131.06927(3)	85
0.87	**Citric acid**	C_6_H_7_O_7_^−^	191.01920	191.01841	0.79	**111.00557(100)**, 112.00888(7)	86
0.60	**Quinic acid**	C_7_H_11_O_6_^−^	191.05560	191.04957	6.03	108.01788(36), 109.02672(85), 110.02807(4), **111.00533(100)**, 112.03631(6), 113.02026(9), 127.03732(60), 137.01908(12), 171.02481(7), 173.0456(6), 191.0522(81), 192.05676(6)	87

**Table 4:** Quantification of phenolic compounds and their derivatives (mg/100 g) in different floral apple pollen samples using UHPLC Q-ToF MS.

Compound Name	POLLEN SAMPLES (mg/100 g FW Pollen)										
	Red Aroma	Discovery	Summerred	Rubinstep	Elstar	Dolgo	Professor Sprenger	Asfari	Eden	Fryd	Katja
***Phenolic acids and derivatives ^a^***											
***Hydroxybenzoic acids and derivatives***											
Hydroxybenzoic acid	<LOQ	12.78	8.03	8.06	<LOQ	<LOQ	<LOQ	<LOQ	<LOQ	<LOQ	14.54
Hydroxybenzoic acid hexoside isomer I	<LOQ	8.90	8.79	13.39	<LOQ	7.40	<LOQ	9.08	<LOQ	<LOQ	<LOQ
Hydroxybenzoic acid hexoside isomer II	31.52	107.24	80.91	33.19	59.72	114.40	63.84	57.23	21.97	27.64	35.17
Dihydroxybenzoic acid hexoside isomer I	-	<LOQ	<LOQ	<LOQ	<LOQ	7.85	16.39	<LOQ	<LOQ	<LOQ	<LOQ
Dihydroxybenzoic acid hexoside isomer II	<LOQ	<LOQ	8.33	7.60	10.90	24.74	34.64	<LOQ	<LOQ	<LOQ	<LOQ
Vanillin	<LOQ	<LOQ	<LOQ	-	<LOQ	<LOQ	<LOQ	<LOQ	<LOQ	<LOQ	<LOQ
Vanilloside	<LOQ	<LOQ	<LOQ	<LOQ	<LOQ	<LOQ	<LOQ	<LOQ	<LOQ	<LOQ	<LOQ
Vanilloloside	-	<LOQ	-	-	-	<LOQ	-	<LOQ	<LOQ	<LOQ	<LOQ
Vanillic acid hexoside isomer I	13.45	15.97	13.61	20.70	7.98	14.81	7.45	13.72	6.86	7.31	6.88
Vanillic acid hexoside isomer II	27.12	29.14	26.46	53.43	28.15	26.10	16.59	53.34	12.85	15.57	16.93
Gallic acid hexoside	<LOQ	<LOQ	<LOQ	<LOQ	<LOQ	7.88	19.09	<LOQ	<LOQ	<LOQ	<LOQ
Syringic acid hexoside	8.40	<LOQ	8.55	16.16	8.14	8.65	<LOQ	11.60	<LOQ	<LOQ	<LOQ
**∑**	**80.48**	**174.02**	**154.68**	**152.53**	**114.88**	**211.81**	**158.00**	**144.97**	**41.67**	**50.52**	**73.52**
***Hydroxycinnamic acids and derivatives***											
3,4-Dimethoxycinnamic acid	67.20	76.25	20.33	45.82	60.10	84.21	20.55	68.17	42.13	71.63	39.97
Coumaric acid hexoside	49.95	51.69	45.29	9.25	52.68	55.08	23.63	39.79	30.66	49.39	46.93
Coumaroylquinic acid isomer I	16.34	15.75	17.68	28.46	26.64	11.27	19.01	18.13	16.57	13.47	10.56
Coumaroylquinic acid isomer II	31.46	34.84	45.19	41.57	53.21	41.42	43.02	72.04	46.90	27.67	34.09
Caffeic acid	9.78	-	8.36	7.99	9.51	7.05	17.41	11.44	-	8.26	-
Caffeoyl deoxytetronic acid	-	-	<LOQ	<LOQ	<LOQ	<LOQ	<LOQ	-	<LOQ	-	<LOQ
Caffeic acid hexoside	48.90	63.30	53.06	68.17	61.17	65.44	74.93	100.08	57.99	74.49	43.97
Dicaffeoyl hexoside	<LOQ	<LOQ	<LOQ	<LOQ	<LOQ	<LOQ	<LOQ	<LOQ	<LOQ	12.33	<LOQ
Caffeoylquinic acid isomer I	69.65	90.24	45.00	102.25	47.86	87.19	103.95	75.56	57.88	23.53	34.57
Caffeoylquinic acid isomer II	93.44	107.56	84.61	94.11	94.88	102.10	93.78	99.43	82.25	51.02	75.28
Dicaffeoylquinic acid	21.30	28.13	26.64	22.57	21.25	17.68	18.66	50.24	35.80	16.84	34.67
Coumaroyl-caffeoylquinic acid	8.22	8.65	9.32	13.16	10.19	9.21	16.42	15.55	12.93	<LOQ	11.31
Ferulic acid hexoside isomer I	-	14.64	14.16	18.21	10.47	11.96	10.96	18.45	9.45	18.86	9.40
Ferulic acid hexoside isomer II	11.22	10.54	<LOQ	-	-	-	-	11.77	20.20	-	12.23
Diferuloyl hexoside isomer I	11.55	12.60	12.61	<LOQ	7.98	9.31	9.52	14.31	<LOQ	12.03	-
Diferuloyl hexoside isomer II	17.03	21.75	15.31	25.12	16.44	22.75	7.32	19.88	18.98	10.59	27.66
Feruloylquinic acid	8.18	16.24	19.14	12.17	6.50	39.34	48.14	14.16	40.03	<LOQ	19.55
Feruloylcaffeic acid hexoside	14.23	11.16	<LOQ	15.17	8.70	18.43	<LOQ	13.30	10.63	14.02	7.61
Sinapic acid hexoside	-	<LOQ	<LOQ	<LOQ	<LOQ	<LOQ	<LOQ	<LOQ	<LOQ	<LOQ	<LOQ
∑	**478.44**	**563.35**	**416.71**	**504.03**	**487.59**	**582.44**	**507.31**	**642.32**	**482.40**	**404.15**	**407.79**
**∑∑**	**558.92**	**737.37**	**571.38**	**656.55**	**602.47**	**794.25**	**665.31**	**787.29**	**524.07**	**454.66**	**481.30**
***Flavonol aglycones and glycosides ^b^***											
***Kaempferol and derivatives***											
Kaempferol	<LOQ	<LOQ	12.55	7.55	<LOQ	<LOQ	<LOQ	<LOQ	15.93	6.09	6.30
Kaempferol-3-*O*-rhamnoside	16.75	8.14	14.07	25.65	5.84	27.06	7.73	<LOQ	24.35	9.93	17.50
Kaempferol 3-*O*-(2″-caffeoyl)-pentoside	<LOQ	5.49	<LOQ	10.10	<LOQ	<LOQ	<LOQ	18.29	10.36	<LOQ	<LOQ
**∑**	**16.75**	**13.62**	**26.61**	**43.30**	**5.84**	**27.06**	**7.73**	**18.29**	**50.64**	**16.03**	**23.79**
***Quercetin and derivatives***											
Quercetin-3-*O*-pentoside	31.32	34.70	38.34	40.52	33.30	49.83	55.10	40.28	31.89	24.06	29.31
Quercetin-3-*O*-rhamnoside	33.60	16.51	22.94	27.14	16.63	35.10	20.77	11.52	13.92	17.19	10.82
Quercetin-3-*O*-hexoside	18.16	20.83	33.99	45.53	27.48	50.64	80.80	29.16	25.67	16.30	20.62
Quercetin-3-*O*-(2″-*O*-malonyl)-hexoside	<LOQ	<LOQ	-	<LOQ	<LOQ	12.65	16.25	-	-	<LOQ	-
**∑**	**83.09**	**72.04**	**95.27**	**113.19**	**77.41**	**148.22**	**172.92**	**80.96**	**71.47**	**57.54**	**60.74**
***Isorhamnetin and derivatives***											
Isorhamnetin	22.08	17.75	65.82	19.08	11.95	<LOQ	28.34	-	70.86	37.53	78.54
Isorhamnetin-3-*O*-hexoside	54.20	-	-	-	54.72	37.28	53.36	-	74.08	-	-
Isorhamnetin-3-*O*-(2″-*O*-malonyl)hexoside	50.95	59.88	61.57	61.64	54.36	29.00	71.70	32.72	69.01	81.03	82.36
Isorhamnetin-3-*O*-(2″-*O*-rhamnosyl)-hexoside	-	14.64	11.28	<LOQ	-	<LOQ	5.67	5.61	28.54	25.31	15.56
Isorhamnetin-3-*O*-(2″-*O*-hexosyl)-hexoside	29.08	44.14	36.05	49.51	23.79	7.97	17.65	23.44	53.84	84.52	68.51
Isorhamnetin-3-*O*-(2″-rhamnosyl-6″-malonyl)-hexoside	-	-	-	-	-	<LOQ	-	<LOQ	<LOQ	<LOQ	<LOQ
Isorhamnetin-3-*O*-(2″-hexosyl-6″-malonyl)-hexoside	34.45	42.55	27.02	57.24	20.46	19.27	26.34	17.10	46.46	65.58	69.06
**∑**	**190.75**	**178.96**	**201.74**	**187.48**	**165.28**	**93.53**	**203.06**	**78.87**	**342.78**	**293.98**	**314.03**
***Syringetin and derivatives***											
Syringetin	20.42	19.78	73.95	12.80	28.03	10.27	25.52	18.14	72.17	25.32	107.59
Syringetin-3-*O*-hexoside	10.76	6.20	15.23	7.31	16.04	5.53	20.18	<LOQ	16.06	43.64	34.35
Syringetin-3-*O*-(6″-*O*-acetyl)-hexoside	-	-	-	-	43.81	-	-	-	-	23.26	30.76
Syringetin-3-*O*-(2″-*O*-rhamnosyl)-hexoside	12.82	9.75	17.41	<LOQ	20.26	14.57	7.11	23.41	20.40	18.00	16.31
Syringetin-3-*O*-(2″-*O*-hexosyl)-hexoside	-	-	<LOQ	<LOQ	-	-	-	-	7.09	10.03	13.42
Syringetin-3-*O*-(2″-rhamnosyl-6″-malonyl)-hexoside	<LOQ	<LOQ	-	-	<LOQ	<LOQ	<LOQ	<LOQ	8.45	5.67	9.37
Syringetin-3-*O*-(6″-hexosyl-6″-malonyl)-hexoside	-	<LOQ	13.20	<LOQ	13.44	-	<LOQ	<LOQ	<LOQ	<LOQ	<LOQ
∑	**44.01**	**35.74**	**119.80**	**20.10**	**121.58**	**30.38**	**52.82**	**41.56**	**124.17**	**125.91**	**211.79**
**∑∑**	**334.59**	**300.37**	**443.42**	**364.06**	**370.12**	**299.18**	**436.53**	**219.67**	**589.07**	**493.46**	**610.36**
***Dihydrochalcone and derivatives ^b^***											
Phloretin	7.98	43.18	49.68	7.81	28.87	31.83	111.20	56.70	67.07	16.77	10.86
Phlorizin	87.55	85.21	87.57	59.54	62.21	57.53	72.39	68.67	109.64	102.99	115.81
Phloretin-4′-*O*-(6″-benzoyl)-hexoside	-	-	-	-	-	-	18.39	-	-	-	-
Phloretin-4′-*O*-(6″-cinnamoyl)-hexoside	<LOQ	<LOQ	-	-	<LOQ	-	10.14	-	-	-	-
Phloretin-2′-*O*-(6″-pentosyl)-hexoside	-	32.28	16.94	46.33	11.87	16.46	12.15	7.69	-	10.52	-
Phloretin-4′-*O*-(6″-caffeoyl)-hexoside	<LOQ	5.70	<LOQ	-	<LOQ	<LOQ	26.54	7.15	<LOQ	<LOQ	<LOQ
Phloretin-4′-*O*-(6″-coumaroyl)-hexoside	10.99	17.50	<LOQ	14.04	18.45	8.51	50.63	15.69	6.82	<LOQ	5.96
Phloretin-4′-*O*-(6″-feruloyl)-hexoside	36.59	34.93	8.72	36.04	54.01	33.97	57.18	59.01	32.46	19.84	23.75
3-Hydroxyphloretin	<LOQ	<LOQ	<LOQ	-	<LOQ	-	9.33	<LOQ	<LOQ	<LOQ	-
3-Hydroxyphloretin-2′-*O*-hexoside	27.40	28.43	27.18	18.74	33.91	-	12.32	-	28.87	24.04	23.47
3-Hydroxyphloretin-2′-*O*-(6″-pentosyl)-hexoside	15.43	19.86	<LOQ	8.04	<LOQ	<LOQ	<LOQ	<LOQ	<LOQ	<LOQ	8.74
**∑**	**185.95**	**267.08**	**190.10**	**206.67**	**209.31**	**148.29**	**380.27**	**214.91**	**244.86**	**174.16**	**188.59**
***Flavanone and Flavan-3-ols ^b^***											
Naringenin	<LOQ	<LOQ	<LOQ	<LOQ	<LOQ	<LOQ	<LOQ	5.92	5.37	9.17	11.79
Epicatechin	<LOQ	17.87	14.56	9.64	5.38	18.58	43.52	15.77	6.53	10.91	<LOQ
**∑**	**-**	**17.87**	**14.56**	**9.64**	**5.38**	**18.58**	**43.52**	**21.69**	**11.90**	**20.08**	**11.79**
**∑∑∑**	**1079.45**	**1322.69**	**1219.46**	**1236.93**	**1187.28**	**1260.30**	**1525.63**	**1243.56**	**1369.91**	**1142.36**	**1292.03**

Abbreviations: “-”—nonidentified compounds. All phenolic acids and derivatives are expressed as gentisic acid equivalents ^a^; all flavonoids and their derivatives are expressed as quercetin equivalents ^b^; “LOQ”—limit of quantification.

**Table 5:** The relative polypeptide composition (%) of extractable proteins in different floral apple pollen samples.

kDa (Ranges)	No. Band	Pollen Samples (%)										
		Red Aroma	Discovery	Summerred	Rubinstep	Elstar	Dolgo	Professor Sprenger	Asfari	Eden	Fryd	Katja
**>95 kDa**	1	0.72	0.55	0.69	0.46	-	1.53	0.95	-	-	-	-
	2	1.36	1.26	1.69	1.96	1.16	1.98	2.17	1.99	1.66	1.70	1.83
	3	-	0.89	1.15	0.49	0.83	1.16	1.23	0.84	1.09	-	-
**∑**		**2.09**	**2.70**	**3.53**	**2.90**	**1.99**	**4.67**	**4.35**	**2.83**	**2.74**	**1.70**	**1.83**
**95–66 kDa**	4	-	-	0.82	-	-	-	-	-	0.98	-	-
	5	0.68	0.80	0.78	0.92	1.35	1.52	1.97	1.12	1.07	1.51	0.92
	6	0.92	1.08	1.17	1.61	0.97	1.00	1.73	1.67	1.46	1.41	1.16
	7	1.83	1.79	2.86	2.05	2.09	2.22	3.12	2.46	2.58	2.26	2.15
**∑**		**3.42**	**3.67**	**5.63**	**4.58**	**4.42**	**4.75**	**6.81**	**5.25**	**6.08**	**5.17**	**4.23**
**66–52 kDa**	8	0.83	0.95	1.06	-	0.82	-	0.95	0.82	1.42	1.54	1.42
	9	-	-	1.14	-	1.34	1.64	1.36	1.15	0.91	1.11	1.43
	10	-	-	1.40	-	-	-	-	-	0.89	-	-
	11	1.57	1.11	1.49	-	1.41	-	1.90	1.46	2.05	1.28	-
	12	5.15	2.77	4.62	3.31	4.09	3.82	3.90	3.29	3.20	3.76	3.56
	13	-	1.20	0.95	0.79	-	-	1.58	1.05	1.64	-	1.06
**∑**		**7.55**	**6.02**	**10.67**	**4.10**	**7.66**	**5.46**	**9.69**	**7.78**	**10.10**	**7.68**	**7.46**
**52–37 kDa**	14	2.00	0.77	2.33	-	1.31	-	1.59	1.28	1.04	1.86	0.73
	15	2.70	2.85	3.06	2.09	2.13	2.05	2.37	2.48	2.38	3.41	1.94
	16	1.96	1.92	2.69	2.54	2.23	2.13	2.63	2.39	2.23	2.40	1.85
	17	1.86	1.87	1.24	1.32	1.36	-	1.70	1.41	1.94	1.59	1.40
**∑**		**8.53**	**7.42**	**9.32**	**5.95**	**7.03**	**4.19**	**8.29**	**7.57**	**7.58**	**9.26**	**5.92**
**37–30 kDa**	18	1.58	1.76	2.27	1.94	2.00	1.33	1.92	2.38	1.66	3.00	1.84
	19	0.99	1.16	0.87	1.07	1.12	1.28	1.36	1.58	1.26	1.34	1.30
	20	1.92	2.48	2.37	2.24	1.61	1.96	2.11	2.36	1.96	2.33	2.59
	21	3.41	1.97	1.96	2.25	2.23	2.11	2.55	2.11	3.08	2.02	1.61
	22	2.06	2.18	5.59	3.01	2.90	3.59	3.25	3.34	4.32	4.32	2.90
	23	-	1.58	1.39	-	0.97	-	-	-	1.21	-	-
**∑**		**9.96**	**11.13**	**14.45**	**10.50**	**10.84**	**10.28**	**11.19**	**11.77**	**13.49**	**13.01**	**10.25**
**30–16 kDa**	24	-	1.15	2.16	-	1.62	-	1.91	1.56	1.38	1.05	1.08
	25	3.41	3.78	3.08	3.78	4.19	3.07	2.76	3.71	3.32	4.36	3.66
	26	-	1.46	2.32	-	-	2.29	-	1.58	1.53	-	2.06
	27	-	0.98	1.44	-	1.41	2.81	5.84	1.94	1.21	1.57	-
	28	7.16	8.23	2.76	8.71	10.49	2.23	-	7.43	3.14	3.02	9.68
	29	-	1.68	2.73	1.52	-	2.28	3.08	2.34	4.54	12.18	2.33
	30	7.66	9.21	4.93	8.25	8.32	8.22	6.74	7.78	5.19	-	7.93
	31	5.40	4.62	5.04	6.40	6.66	7.00	5.64	5.50	5.90	7.67	7.66
	32	6.95	5.34	5.44	4.80	6.05	3.43	5.51	5.18	5.34	6.52	5.34
**∑**		**30.58**	**36.45**	**29.90**	**33.45**	**38.74**	**31.35**	**31.48**	**37.03**	**31.55**	**36.37**	**39.74**
**16–6.5 kDa**	33	4.77	3.74	3.47	2.76	1.61	4.65	3.39	2.54	3.42	3.31	2.77
	34	11.46	9.86	6.42	11.06	9.47	10.37	8.18	8.91	9.00	11.43	11.48
	35	5.50	4.05	6.41	5.12	4.18	4.97	4.83	2.42	3.19	3.17	2.28
	36	4.81	4.20	4.02	3.97	4.12	5.96	3.77	3.69	3.67	2.81	3.50
**∑**		**26.55**	**21.85**	**20.33**	**22.91**	**19.38**	**25.95**	**20.17**	**17.55**	**19.28**	**20.71**	**20.03**
**<6.5 kDa**	37	4.21	3.56	1.15	4.20	2.40	-	1.77	3.05	2.37	-	2.51
	38	7.10	5.57	3.40	6.87	5.75	9.28	5.25	5.99	5.42	6.10	7.13
	39	-	1.62	1.62	4.54	1.78	4.07	1.00	1.18	1.38	-	0.89
**∑**		**11.31**	**10.76**	**6.17**	**15.62**	**9.93**	**13.36**	**8.02**	**10.23**	**9.17**	**6.10**	**10.53**
**∑∑**		**100**	**100**	**100**	**100**	**100**	**100**	**100**	**100**	**100**	**100**	**100**

“-”—band not detected.

## Data Availability

Data are contained within the article.

## Supplementary Materials

The following supporting information can be downloaded at https://www.mdpi.com/article/10.3390/antiox13111374/s1, Table S1. The equation parameters and correlation coefficient (R^2^) of the used phenolic standards for quantification. Table S2. The relative content (%) of phenylamides in different floral apple pollen samples, using UHPLC Q-ToF MS. Table S3. The number of principal components and the percentage of variance they explain. Figure S1. The chromatographic separation of the sugars in the pollen of the apple cultivar Elstar.
