# Supplementary material for: Diversity and Chemical Characterization of Apple (Malus sp.) Pollen: High Antioxidant and Nutritional Values for Both Humans and Insects
Source: Antioxidants (Basel). 2024 Nov 9;13(11):1374. doi: 10.3390/antiox13111374 (PMC11591099; doi:10.3390/antiox13111374)
Supplement: Supplementary file 1 [file antioxidants-13-01374-s001.zip › antioxidants-3250743-supplementary.pdf]

Article

Diversity and Chemical Characterization of Apple (*Malus* sp.) Pollen: High Antioxidant and Nutritional Values for Both Humans and Insects

Milica M. Fotirić Akšić [1], Mirjana B. Pešić [1], Ilinka Pećinar [1], Aleksandra Dramićanin [2], Danijel D. Milinčić [1], Aleksandar Ž. Kostić [1], Uroš Gašić [3], Mihajlo Jakanovski [4], Marko Kitanović [1] and Mekjell Meland [5,*]

[1] Faculty of Agriculture, University of Belgrade, Nemanjina 6, 11000 Belgrade, Serbia; fotiric@agrif.bg.ac.rs (M.M.F.A.); mpesic@agrif.bg.ac.rs (M.B.P.); ilinka@agrif.bg.ac.rs (I.P.); danijel.milincic@agrif.bg.ac.rs (D.D.M.); akostic@agrif.bg.ac.rs (A.Ž.K.); marko.kitanovic@agrif.bg.ac.rs (M.K.)
[2] Faculty of Chemistry, University of Belgrade, Studentski Trg 12-16, 11000 Belgrade, Serbia; akosovic@chem.bg.ac.rs
[3] Institute for Biological Research "Siniša Stanković",, University of Belgrade, Bulevar Despota Stefana 142, 11060 Belgrade, Serbia; uros.gasic@ibiss.bg.ac.rs
[4] Innovative Centre of the Faculty of Chemistry, University of Belgrade, Studentski Trg 12-16, 11000 Belgrade, Serbia; jakanovski@chem.bg.ac.rs
[5] Norwegian Institute of Bioeconomy Research—NIBIO Ullensvang, Ullensvangvegen 1005, 5781 Lofthus, Norway
* Correspondence: mekjell.meland@nibio.no; Tel.: +47-906-28-538

Table S1. Equation parameters and correlation coefficient (R^2) of used phenolic standards for quantification

**Standards**	**Y=a*X±b**	**R^2**	**Linar range (ppm)**	**LOD (ppm)**	**LOQ (ppm)**
Gentisic acid	Y=1726721.3256*X+466061.0629	0.9924	0.1-10	0.99	3.33
Quercetin	Y=769214.5670*X+825343.6827	0.9915	0.1-7	0.79	2.65

Table S2. Relative content (%) of phenylamides in different floral apple pollen samples, using UHPLC Q-ToF MS.

Compound name	Apple pollen samples (%)										
	Red Aroma	Discovery	Summer red	Rubinstep	Elstar	Dolgo	Professor Sprenger	Asfari	Eden	Fryd	Katja
Hydroxycinnamic acid amides											
Putrescine derivatives											
**Coumaroyl putrescine isomer I**	0.41	0.29	0.37	0.46	0.47	0.62	1.18	0.74	1.15	-	0.33
**Coumaroyl putrescine isomer II**	9.55	7.53	6.66	7.57	8.12	10.26	10.89	8.25	8.42	8.49	9.42
**Acetyl coumaroyl putrescine**	1.60	0.47	0.55	0.76	0.89	0.95	0.75	0.46	0.78	0.96	1.03
**Dicoumaroyl putrescine**	15.96	23.42	19.26	20.25	20.41	17.62	23.41	22.40	20.17	19.36	21.11
**Caffeoyl putrescine**	1.41	0.57	0.67	0.33	0.83	0.89	1.05	0.87	0.63	0.44	0.32
**Coumaroyl caffeoyl putrescine**	4.45	5.02	5.43	3.56	4.56	3.83	5.77	5.87	3.65	2.35	2.97
**Dicaffeoyl putrescine**	1.07	1.58	2.13	1.43	1.77	2.26	1.88	2.66	0.98	0.65	0.53
**Feruloyl putrescine**	0.75	0.83	1.11	0.90	1.14	0.87	-	1.29	0.79	0.92	0.59
**Coumaroyl feruloyl putrescine**	0.81	1.89	1.76	1.09	-	1.87	1.13	2.50	-	1.19	-
∑	**36.00**	**41.61**	**37.94**	**36.37**	**38.19**	**39.17**	**46.06**	**45.04**	**36.59**	**34.36**	**36.30**
Spermidine derivatives											
**Coumaroyl spermidine**	1.63	1.54	0.96	2.69	1.79	1.52	0.60	1.18	1.58	0.80	1.05
**Dicoumaroyl spermidine isomer II**	1.58	1.60	1.55	2.20	1.86	1.55	1.36	1.84	2.09	2.19	2.14
**Dicoumaroyl spermidine isomer I**	22.39	18.58	22.25	20.67	15.98	20.13	16.50	18.92	24.96	20.68	19.09
**Tricoumaroyl spermidine**	28.00	28.63	24.67	28.68	29.62	25.39	28.06	19.63	23.39	31.10	32.11
**Coumaroyl caffeoyl spermidine**	2.23	1.43	3.06	1.05	1.80	0.73	0.93	2.04	1.23	0.91	0.45
**Dicoumaroyl caffeoyl spermidine**	3.87	3.57	5.20	3.82	5.32	5.58	4.30	4.95	3.82	3.34	4.43
**Diferuloyl spermidine**	0.16	0.14	0.09	0.41	0.08	0.27	-	0.41	0.40	0.61	0.41
**Coumaroyl feruloyl spermidine**	3.14	2.02	2.43	3.04	3.40	4.46	1.32	2.55	4.61	4.52	2.73
**Dicoumaroyl feruloyl spermidine**	0.99	0.87	1.85	1.07	1.96	1.19	0.86	3.43	1.33	1.49	1.30
∑	**64.00**	**58.39**	**62.06**	**63.63**	**61.81**	**60.83**	**53.94**	**54.96**	**63.41**	**65.64**	**63.70**

Abbreviations: The relative content (%) of individual phenylamides in each pollen sample calculated as the ratio of areas of individual and total phenylamides (share of total area is defined as 100% for each sample); "-" non-identified compounds.

Table S3. The number of principal components and the percentage of variance they explain.

Principal Component Number	Eigenvalue of Cov(X)	% Variance Captured	% Variance Captured Total
1	2.05e+001	31.60	31.60
2	1.22e+001	18.74	50.34
3	6.44e+000	9.90	60.24
4	5.73e+000	8.82	69.06
5	5.25e+000	8.08	77.14
6	4.54e+000	6.98	84.13
7	3.43e+000	5.28	89.41

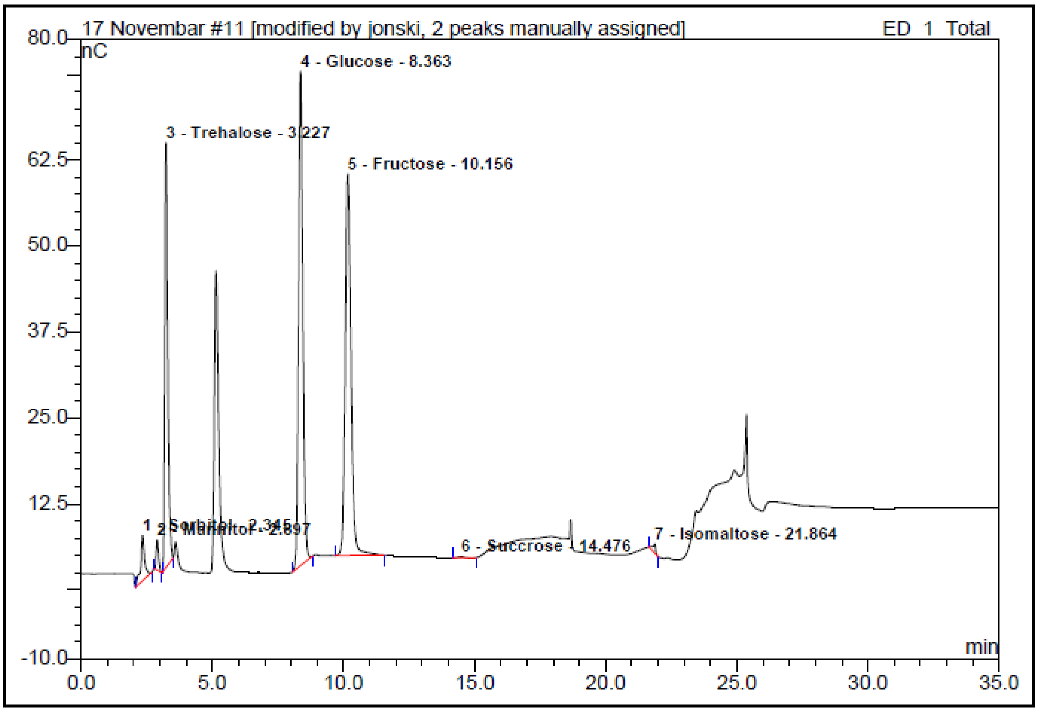

Figure S1. Chromatographic separation of sugars in pollen of the apple cultivar Elstar
